# Artificial sweeteners inhibit multidrug‐resistant pathogen growth and potentiate antibiotic activity

**DOI:** 10.15252/emmm.202216397

**Published:** 2022-11-22

**Authors:** Rubén de Dios, Chris R Proctor, Evgenia Maslova, Sindija Dzalbe, Christian J Rudolph, Ronan R McCarthy

**Affiliations:** ^1^ Division of Biosciences, Department of Life Sciences, Centre of Inflammation Research and Translational Medicine, College of Health, Medicine and Life Sciences Brunel University London Uxbridge UK; ^2^ Division of Biosciences, Department of Life Sciences, Centre for Genome Engineering and Maintenance, College of Health, Medicine and Life Sciences Brunel University London Uxbridge UK

**Keywords:** *Acinetobacter baumannii*, antimicrobial, artificial sweetener, biofilm, carbapenem, Microbiology, Virology & Host Pathogen Interaction, Pharmacology & Drug Discovery

## Abstract

Antimicrobial resistance is one of the most pressing concerns of our time. The human diet is rich with compounds that alter bacterial gut communities and virulence‐associated behaviours, suggesting food additives may be a niche for the discovery of novel anti‐virulence compounds. Here, we identify three artificial sweeteners, saccharin, cyclamate and acesulfame‐K (ace‐K), that have a major growth inhibitory effect on priority pathogens. We further characterise the impact of ace‐K on multidrug‐resistant *Acinetobacter baumannii*, demonstrating that it can disable virulence behaviours such as biofilm formation, motility and the ability to acquire exogenous antibiotic‐resistant genes. Further analysis revealed the mechanism of growth inhibition is through bulge‐mediated cell lysis and that cells can be rescued by cation supplementation. Antibiotic sensitivity assays demonstrated that at sub‐lethal concentrations, ace‐K can resensitise *A. baumannii* to last resort antibiotics, including carbapenems. Using a novel *ex vivo* porcine skin wound model, we show that ace‐K antimicrobial activity is maintained in the wound microenvironment. Our findings demonstrate the influence of artificial sweeteners on pathogen behaviour and uncover their therapeutic potential.

## Graphical abstract

Artificial sweeteners can inhibit the growth of multidrug resistant pathogens, disrupt virulence associated behaviours and potentiate antibiotic activity. In wound models the sweetener, Acesulfame K, shows potential as a topical wound treatment.

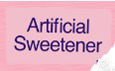

## Introduction

The discovery of penicillin marked the beginning of a golden age in antibiotic discovery, with new classes of antibiotics being routinely discovered. However, since the beginning of the 1990s, the rate of antibiotic discovery has slowed to a near standstill (Hutchings *et al*, 2019). This lack of discovery, compounded by the rapid emergence and spread of bacterial pathogens that exhibit resistance to first‐line antibiotic treatments, has led to an antibiotic resistance crisis worldwide (Lewis, 2020). Deaths attributed to antimicrobial resistance (AMR) reached 4.95 million in 2019 (Murray *et al*, 2022), and a predicted cumulative global cost of $100 trillion by 2050 (HM Goverment, 2019). A 2018 report from the World Health Organisation (WHO) placed *Acinetobacter baumannii* and *Pseudomonas aeruginosa* at the top of a global priority list of bacteria in urgent need of novel therapeutic interventions. These organisms are often resistant to many commonly used antibiotics, even developing resistance against several last‐line antibiotics such as colistin, carbapenems and tigecycline (Cai *et al*, 2017; Andrade *et al*, 2020; Hua *et al*, 2021). With the continued rise of antibiotic resistance and the emergence of multidrug‐resistant (MDR), extensively drug‐resistant and pan‐resistant pathogens, the need to identify new compounds with antibiotic properties is more urgent than ever. This has prompted scientists to explore new environments and approaches to identify potential therapeutic agents. However, new drug development is associated with significant financial and time commitments, with the average new drug taking up to 20 years and $1.33 billion to bring to market (Wouters *et al*, 2020; Brown *et al*, 2022). The high cost and long‐time frame, combined with the high failure rate of novel active pharmaceutical agents in the development pipeline, make it clear that alternative methods are required. One of the most popular approaches in recent years has been mining for new chemical compounds in understudied niches. Many studies have investigated natural reservoirs, such as marine and terrestrial plants, to identify new antimicrobial compounds. Several phytochemicals, such as coumarin, cinnamaldehyde and baicalin, have been identified as potential antimicrobial or anti‐virulence therapies (Gutiérrez‐Barranquero *et al*, 2015; Slachmuylders *et al*, 2018; Ahmed *et al*, 2019). Interestingly, it has been noted that phytochemicals such as these, when present in the diet, may impact processes like bacterial signalling and communication in the gut. Further, it has been suggested that these compounds may be used to treat and control pathogenic organisms (McCarthy & O'Gara, 2015; Proctor *et al*, 2020). With such a wide array of dietary phytochemicals showing antimicrobial and anti‐virulence activity, the assessment of non‐phytochemical dietary compounds as potential therapeutics is an obvious next step. While there are many kinds of food additives and other compounds that may be investigated, artificial sweeteners are one of the most ubiquitous.

Artificial sweeteners (AS), also known as non‐nutritive or non‐caloric sweeteners, are compounds that demonstrate significantly higher sweetening power when compared to sucrose. Their caloric contribution is negligible or zero, and they are most commonly used for their sweetening function only (Carocho *et al*, 2017). Some of the most popular intensive sweeteners include aspartame, saccharin, sucralose, and acesulfame‐K (ace‐K). Although AS were first discovered in the 19th century with the synthesis of saccharin (Tandel, 2011), they remain a subject of controversy. Today, AS are simultaneously described as one of the most important achievements in the food industry to date and presented as poorly understood substances with unknown effects on human health (Carocho *et al*, 2017). Despite this, the alarming increase in global obesity has led to increased research into the impact of AS on human health and our environment. Several studies have noted that AS are an emerging source of pollution in the environment (Scheurer *et al*, 2009; Naik *et al*, 2021) as it has been shown that a significant proportion of AS move through the gastrointestinal (GI) tract unchanged or being degraded only slightly (Roberts *et al*, 2000), thus being expelled into wastewater. Recent work has shown that the levels of AS present in wastewater can influence the behaviour of environmental bacteria, promoting lateral gene transfer (i.e. conjugation and natural transformation), potentially facilitating the exchange of associated antibiotic‐resistant genes (Yu *et al*, 2021, 2022). This finding clearly highlights that some AS possess a biological activity on bacteria. This has led researchers to investigate the effect of AS on bacterial communities in the body, such as the gut microbiome.

Interestingly, a variety of evidence exists on the impact of AS on bacterial populations in the gut. A recent study by Markus *et al* (2021) suggested that sucralose has a significant inhibitory effect on quorum sensing in Gram‐negative bacteria, consequently disturbing the balance of the GI microbial community. Similarly, a separate study has demonstrated that the consumption of AS, including sucralose, induces functional and compositional changes in the mouse microbiome, leading to glucose intolerance (Suez *et al*, 2014). Suez *et al* (2022) reported that different AS altered the stool and oral microbiome. The authors also present evidence that these AS‐driven changes in microbiome impact an individual's glycaemic response. While these studies show significant effects of AS on the microbiome and further downstream effects, other studies have shown a more basic, but equally important, effect of AS on individual bacterial species. Ace‐K has been shown to promote the growth of organisms such as *Escherichia coli* in a number of studies (Mahmud *et al*, 2019; Shahriar *et al*, 2020). Conversely, negative impacts on the growth of laboratory *E. coli* strains have been reported (Wang *et al*, 2018). Despite these contradictory findings, the majority of the studies agree that AS do have the potential to alter the composition and/or functions of the microbiome.

The lack of evidence for the activity of AS, as well as the comparative lack of any studies exploring the impact of these compounds on pathogenic organisms, prompted our investigations. Here, we have identified an AS that displays robust antibacterial activity against some of the most prevalent MDR bacterial pathogens (*Enterococcus faecalis*, *Klebsiella pneumoniae*, *A. baumannii*, *P. aeruginosa and Enterobacter cloacae*). These pathogens are the major cause of nosocomial infections and can persist even after being treated with antimicrobial agents (Mulani *et al*, 2019). Using the MDR *A. baumannii* strain AB5075 as a model, we demonstrate that, as well as inhibiting growth, ace‐K in particular is capable of inhibiting a range of virulence behaviours, such as biofilm formation (associated with persistent infection), motility (associated with dissemination of bacteria within the host) and natural transformation (associated to the spread of AMR genes). We use differential RNA sequencing (dRNA‐seq) to uncover the mechanism of action of ace‐K and demonstrate that it causes bulge‐mediated cell lysis and confirm that this mechanism is conserved across species. Remarkably, we also show that ace‐K can potentiate the activity of a range of clinically relevant antibiotics, including carbapenems. Finally, we demonstrate the potential of ace‐K to be repurposed as a topical antimicrobial using *ex vivo* laceration and burn wound models.

## Results

### Artificial sweeteners can negatively impact the growth of MDR pathogens

**Figure 1:**
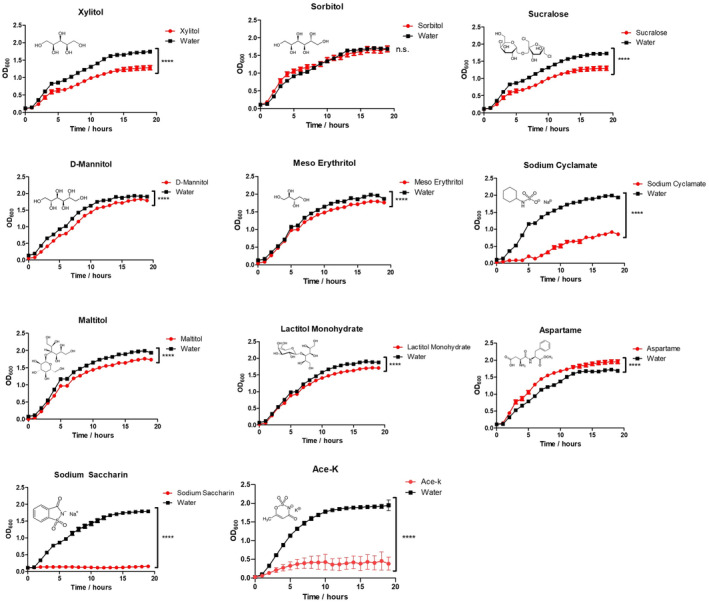
Impact of different AS on *Acinetobacter baumannii* growth kinetics Xylitol, sucralose, D‐mannitol, erythritol, sodium cyclamate, maltitol, lactitol, sodium saccharin and ace‐K at 2.66% concentration all significantly inhibited the growth of MDR *A. baumannii* AB5075 to varying degrees over 19 h of growth. Sodium saccharin, ace‐K and sodium cyclamate had the strongest negative effects while 1% aspartame promoted growth. Sorbitol showed no significant inhibition or promotion of growth in AB5075. Data are the average of three biological replicates ± standard deviation (S.D). Statistical analysis was performed by two‐way repeated‐measures ANOVA (****P ≤ 0.0001).

**Figure 2:**
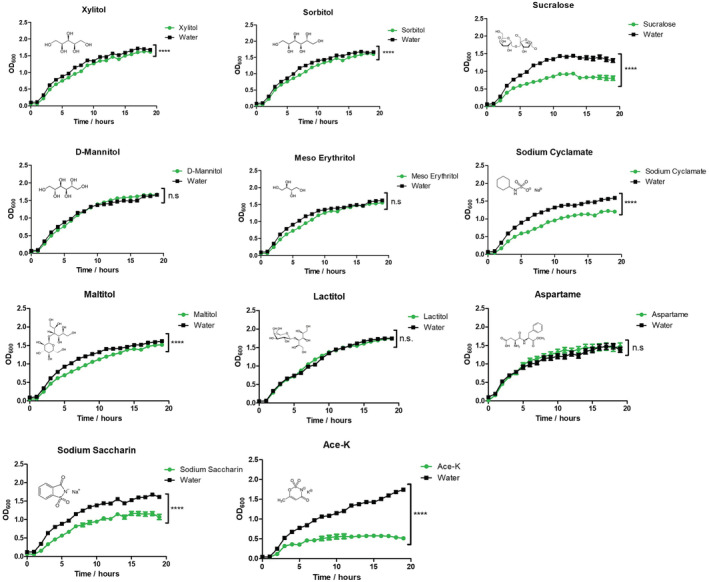
Impact of different AS on *Pseudomonas aeruginosa* PA14 growth kinetics Xylitol, sorbitol, sucralose, erythritol, sodium cyclamate, maltitol, sodium saccharin and ace‐K at 2.66% concentration all significantly inhibited the growth of *P. aeruginosa* PA14 to varying degrees over 19 h growth. Mannitol, lactitol and aspartame showed no effect on growth. Data are the average of three biological replicates ± S.D. Statistical analysis was performed by two‐way repeated‐measures ANOVA (***P ≤ 0.001, ****P ≤ 0.0001).

In order to determine whether different AS could impact the growth of MDR pathogens, the clinical isolate *A. baumannii* AB5075 and lab strain *P. aeruginosa* PA14 were cultured in LB broth supplemented with a selection of the most widely used AS including xylitol, sorbitol, sucralose, D‐mannitol, erythritol, sodium cyclamate, maltitol, lactitol, sodium saccharin, aspartame, or ace‐K. Each sweetener was used at a concentration of 2.66%, as this represented the maximum common concentration at which all sweeteners could be dissolved and ensured the effect of each AS was directly comparable. As aspartame has a significantly lower solubility than the other sweeteners tested, it was used at a concentration of 1%. The majority of these sweeteners had a minor but significant negative impact on the growth of *A. baumannii* (Fig 1). There were some exceptions; sorbitol had no impact on the growth of AB5075 and aspartame significantly increased the growth. *P. aeruginosa* PA14 growth was significantly affected by xylitol, sorbitol, sucralose, sodium cyclamate, maltitol, sodium saccharin and ace‐K at a concentration of 2.66%. No significant impact on growth was seen with mannitol, lactitol or aspartame exposure (Fig 2). Endpoint analysis (after 19 h) of the degree of growth inhibition for each sweetener against AB5075 and PA14 are reported in Appendix Figs S1 and S2, respectively.

Of those AS that had a negative impact on growth the strongest effects were seen for sodium cyclamate, sodium saccharin and ace‐K. However, for sodium cyclamate and sodium saccharin, the strength of the inhibitory effect varied between the pathogens, whereas the effect was consistent between both pathogens for ace‐K, therefore we focused on this sweetener for further characterisation.

### Ace‐K inhibits growth in a dose‐dependent manner and cannot be used as a carbon source

**Figure 3:**
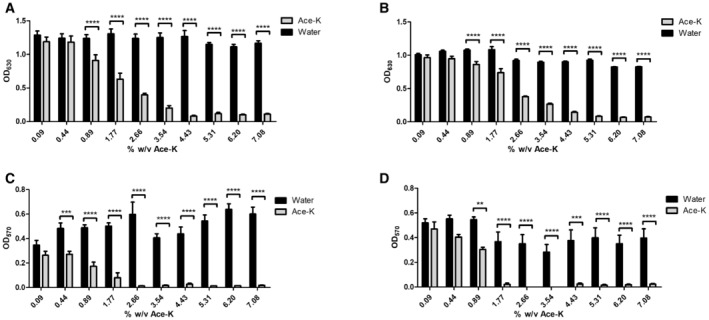
Minimum inhibitory concentration (A, B) and minimum biofilm inhibitory concentration (C, D) of ace‐K against AB5075 (A, C) and PA14 (B, D) A–DSignificant inhibition of AB5075 at 0.89% ace‐K and inhibition of PA14 planktonic growth at 0.44% ace‐K and above. Growth inhibition was shown to be dose‐dependent against both organisms up to a concentration of 5.31% beyond which no further inhibition occurred with increasing concentration. Almost complete inhibition of biofilm formation was achieved at concentrations of 2.66% against AB5075 and 1.77% against PA14. Samples were tested in biological triplicate with technical quadruplets. Data represent the mean of three biological replicates ± S.D. Statistical analysis was by two‐way repeated‐measures ANOVA (*P ≤ 0.05, **P ≤ 0.01, ***P ≤ 0.001, ****P ≤ 0.0001).

We continued to focus on ace‐K by investigating whether its impact on bacterial growth was dose‐dependent. A minimum inhibitory concentration (MIC) assay was performed for both pathogens, with ace‐K concentrations ranging from 0.09 to 7.08%. A significant reduction in growth could be distinguished at 0.89% ace‐K for *A. baumannii*, with the effect plateauing at 4.43% and above (Fig 3A). The half maximal inhibitory concentration (IC_50_) for ace‐k against AB5075 in liquid culture was calculated to be 2.20%. *P. aeruginosa* growth was impacted at 0.44% and reached maximum inhibition at 5.31% and above (Fig 3B). The IC_50_ for ace‐k against PA14 was calculated to be 2.85%. We also explored the ability of either of these pathogens to be able to utilise ace‐K as a carbon source. However, culturing both strains in M9 minimal medium in the presence of 2.66% of ace‐K resulted in no growth after 24 h, indicating that it could not be used as a carbon source (Appendix Fig S3). Altogether, these results indicate that ace‐K has a significant antimicrobial effect on *A. baumannii* AB5075 and *P. aeruginosa* PA14 and that it cannot be used by these organisms to facilitate growth.

### Ace‐K inhibits *A. baumannii* and *P. aeruginosa* biofilm formation

Biofilm formation is linked to up to 80% of hospital‐associated infections and is a major factor in the routine failure of antibiotic therapy (Jamal *et al*, 2018). Since ace‐K showed a significant inhibitory effect on pathogens notorious for their ability to form biofilms in the host (Mulcahy *et al*, 2013; Harding *et al*, 2018; Maslova *et al*, 2021), we assessed the ability of ace‐K to inhibit this recalcitrant behaviour. To determine whether ace‐K altered biofilm formation in *A. baumannii* AB5075 and *P. aeruginosa* PA14, a minimum biofilm inhibitory concentration (MBIC) assay was performed (Fig 3C and D). This experiment revealed that even at a growth sub‐inhibitory concentration of 0.44% ace‐K had a significant negative impact on biofilm formation for *A. baumannii* AB5075. Furthermore, concentrations above 2.66% for *A. baumannii* (Fig 3C) and 1.77% for *P. aeruginosa* (Fig 3D) resulted in complete inhibition of biofilm formation. Together, these findings provide evidence that ace‐K has anti‐virulence properties. As established biofilms are a significant clinical challenge, we assessed the ability of ace‐K to eradicate established biofilms in both AB5075 and PA14. Treatment with 8.85% ace‐K reduced total biofilm biomass in AB5075 and PA14 by 48.8 and 69.7%, respectively (Appendix Fig S4).

### Ace‐K alters global gene expression in *A. baumannii*

**Figure 4:**
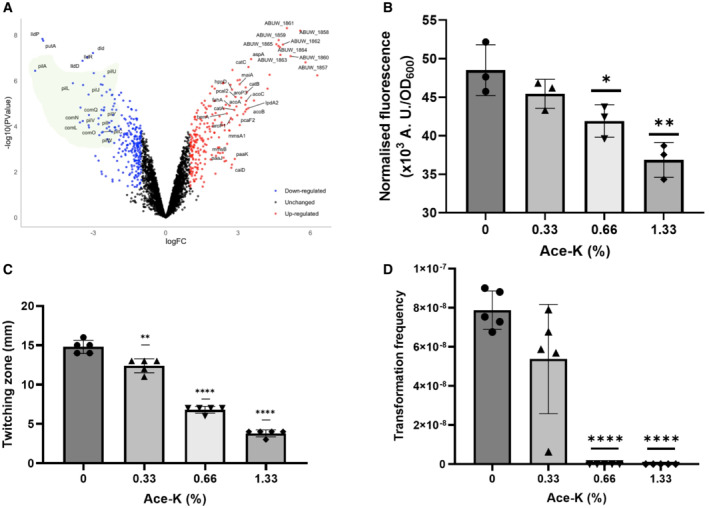
Impact of ace‐K on gene expression and behaviour AVolcano plot representing dRNA‐seq results from comparing global transcription in the presence of ace‐K to a mock treatment. According to the dRNA‐seq results, 212 genes were upregulated (red) and 252 were downregulated (blue) more than 2‐fold in the presence of 1.33% ace‐K compared with water control. Representative genes appear labelled and *pil* and *com* genes, related to twitching motility and natural transformation, are shaded in green (genes with nonsignificant changes in transcription are represented in black).BNormalised fluorescence from the *PpilA::gfpmut3* transcriptional fusion showing the ace‐K mediated downregulation of *pilA* (ABUW_0304). Data represent the mean of three biological replicates each with two technical replicates ± S.D. Analysis is by independent *t*‐tests (*P ≤ 0.05, **P ≤ 0.01).C, DTwitching motility (C) and natural transformation (D) assays show the phenotypic effect of the downregulation of *pil* genes by ace‐K. Different ace‐K concentrations of 0.33, 0.66 and 1.33% were compared with mock treatment. Samples were tested in biological quintuplet for twitching motility and natural transformation. Data represent the mean of five biological replicates ± S.D. Analysis was by independent *t*‐test between treated samples and the corresponding water control (**P ≤ 0.01, ****P ≤ 0.0001).

Considering the effects of ace‐K on growth and biofilm formation observed for *A. baumannii*, a dRNA‐seq analysis was performed to determine the impact of ace‐K exposure on global gene expression patterns. As a condition of the study, we chose an ace‐K concentration that showed an intermediate effect on growth (1.33%) compared with a mock treatment, in order to minimise additional effects on global transcription due to a reduced growth rate or pleiotropic effects. Treated and control cultures were grown to mid‐exponential phase, and their total RNA was extracted, cDNA synthesised, sequenced and compared. As a result, the expression of 464 genes was significantly altered in the presence of ace‐K (212 genes appeared upregulated more than 2‐fold, whereas 252 appeared downregulated; Fig 4A; Dataset EV1). To assess the functional relevance of the significantly regulated genes, we based our analysis on the current AB5075‐UW annotation (Gallagher *et al*, 2015) and a Gene Set Enrichment Analysis (GSEA) performed with FUNAGE‐Pro (de Jong *et al*, 2022; an ordered summary of this analysis can be found in Dataset EV2).

According to their functional GSEA clustering, the dRNA‐seq data highlighted a variety of gene groups with specific functions in the *A. baumannii* physiology, including a significant proportion of genes encoding membrane‐associated proteins, which appeared dysregulated in the presence of ace‐K (Dataset EV3). Among the other functional groups, we observed a general induction of genes related to iron uptake, including the *pfeA* siderophore receptor coding gene (ABUW_2916), *feoA* iron transporter (ABUW_3633) and the *fecIR* iron acquisition σ‐anti‐σ system (ABUW_2986–2987). Consistently, siderophore biosynthesis‐related genes, such as the *bfn* gene cluster, encoding the baumanoferrin biosynthesis pathways (ABUW_2178–2189), as well as *bauF* and *basE* (ABUW_1168 and ABUW_1180, respectively), belonging to the acinetobactin biogenesis pathway (ABUW_1168–1188) were significantly upregulated. Together, this agrees with an upregulation of the *fur* transcriptional regulator in the presence of ace‐K. Since this suggested that ace‐K might be causing iron depletion to the cell, we attempted to relieve the ace‐K effect by adding external iron. However, Fe^2+^ did not rescue cells and Fe^3+^ only produced a very minor recovery that did not improve through increasing iron concentrations (Appendix Fig S5). This suggests that iron does not play a relevant role in the ace‐K antimicrobial mechanism.

Genes coding for Csu pili (ABUW_1487–1492), as well as a Bap (biofilm‐associated protein) coding gene (ABUW_0916), were downregulated in the presence of ace‐K. Csu pili are well known for their essential role in biofilm formation in *A. buamannii* (Moon *et al*, 2017; Pakharukova *et al*, 2018; Romero *et al*, 2022). To validate that this downregulation of the *csu* genes was responsible for the diminished biofilm phenotype, we tested the biofilm forming ability of a number of mutants in the downregulated *csu* genes obtained from the Manoil *A. baumannii* transposon mutant library (Gallagher *et al*, 2015). These mutants exhibited a defect in biofilm formation with respect to the wild type AB5075 (Appendix Fig S6), thus linking their downregulation to the decreased biofilm formation in the presence of ace‐K. Additionally, this downregulation occurs concomitantly with an upregulation of the whole *paa* phenylacetic acid degradation pathway (ABUW_2525–2536). Recent reports have shown a negative correlation between the *paa* and *csu* expression levels upon antibiotic treatment, thus linking phenylacetic acid levels to biofilm formation (Moon *et al*, 2017; Hooppaw *et al*, 2022). This result, together with the MBIC data for *A. baumannii* (Fig 2C), suggests that biofilm inhibition in the presence of ace‐K is caused by the downregulation of the *csu* genes in a phenylacetic acid signalling‐dependent manner.

The most striking observation in the presence of ace‐K is the downregulation of most genes coding for Type 4 Pili (T4P) biogenesis, machinery and regulation. These bacterial appendages are key structures for twitching motility in *A. baumannii* (Harding *et al*, 2013; Chlebek *et al*, 2019; Ellison *et al*, 2021). Furthermore, together with the genes involved in DNA uptake, single‐stranded DNA binding and recombination, T4P plays a key role in natural competence and transformation (Vesel & Blokesch, 2021). This general downregulation includes *pilA*, *fimT, pilVWXY*, *pilGHIJL*, *pilZ*, *pilTU*, *pilBCD* and *pilR* (ABUW_0304, ABUW_0313–0317, ABUW_0678–0682, ABUW_2255, ABUW_3031–3032, ABUW_3549–3551 and ABUW_3641, respectively). Also, a number of genes annotated as *com* (*comMNOLQ* and *comEF*, with locus tags ABUW_0290–0294 and ABUW_0318–0319, respectively) appear downregulated. However, according to the gene description provided with the current AB5075 annotation (AB5075‐UW, Gallagher *et al*, 2015), they should be classified as part of the *pil* clusters, which is further supported by the T4P genomic comparisons presented by Vesel & Blokesch (2021). With respect to the genes directly involved in DNA uptake and stabilisation, only *dprA* (ABUW_3723) appears downregulated.

### Ace‐K abolishes twitching motility and natural transformation in *A. baumannii*

Motility, and twitching in particular for *A. baumannii*, is a central facet of virulence and facilitates bacterial dissemination to the bloodstream or other niches within an infected host (Guoqi *et al*, 2018; Maslova *et al*, 2021). As mentioned above, almost all genes involved in T4P assembly and control of protrusion/retraction dynamics were significantly downregulated in the presence of ace‐K according to our dRNA‐seq data. To validate this regulation, we selected the gene ABUW_0304, encoding the major pilin *pilA* (Ronish *et al*, 2019), which exhibits the greatest downregulation in the presence of ace‐K, and constructed a transcriptional *gfp* fusion to its promoter. Using the fluorescence produced by this *PpilA::gfpmut3* fusion as a readout of *pil* regulation, we set up an assay treating AB5075 with different ace‐K concentrations for 2 h. The ace‐K concentrations used in this assay ranged from 0.33% to that used in our dRNA‐seq experiment (1.33%) and were compared with a mock treatment. As a result, AB5075 bearing the *PpilA::gfpmut3* fusion produced decreasing levels of fluorescence over increasing concentrations of ace‐K, thus validating our dRNA‐seq results (Fig 4B). To further validate this at the phenotypic level, we performed twitching assays using the same range of ace‐K concentrations assayed with the *PpilA::gfpmut3* transcriptional fusion (Fig 4C). In agreement with the dRNA‐seq and fluorimetry results, we observed a dose‐dependent decrease in the twitching motility of AB5075 over increasing concentrations of ace‐K. Furthermore, this effect was not strain‐specific, as other commonly used *A. baumannii* strains (AB0057 and BAA 747) also exhibited a dose‐dependent decrease in twitching motility within the same range of ace‐K concentrations (Appendix Fig S7A and B).

A key finding from two recent studies by Yu *et al*, that explored the influence of ace‐K in wastewater on environmental bacteria, was that those ace‐K concentrations (ranging from 3 × 10^−7^ to 0.03%) could promote lateral gene transfer by increasing conjugation and natural transformation frequency (Yu *et al*, 2021, 2022). Hence, ace‐K may stimulate the acquisition of antibiotic‐resistant genes (Yu *et al*, 2022). However, our transcriptomic data, performed after treatment with higher ace‐K concentrations, show that genes linked to the natural transformation, such as the aforementioned *pil* genes, were downregulated. To address whether this effect on transcription could have a reflection on the phenotype, we tested the impact of the previously mentioned range of ace‐K concentrations on natural transformation in *A. baumannii* AB5075. Remarkably, and in accordance with our transcriptomic data, fluorimetry and pili‐mediated motility results, transformation frequency was impacted by ace‐K, with transformation being completely abolished at 0.66% and above (Fig 4D). Furthermore, the drop in transformation frequency occurred even in the presence of divalent cations (2 mM CaCl_2_ and 1 mM MgSO_4_), which are proven to increase natural transformation frequency in *A. baumannii* (Traglia *et al*, 2016; Appendix Fig S7C). In this condition, a significant drop‐in transformability was observed at 0.33% compared with the control, reaching transformation abolition at 0.66% ace‐K and above. Also, as cell viability was not affected in the presence of cations when supplementing with ace‐K (Appendix Fig S7D), we discarded the possibility that this effect might be due to growth inhibition.

Altogether, these findings add to the repertoire of anti‐virulence properties associated with ace‐K and indicate that, above certain concentrations, it could actually limit the acquisition of antimicrobial resistance elements in pathogenic bacteria.

### Ace‐K has broad‐spectrum antimicrobial activity

**Figure 5:**
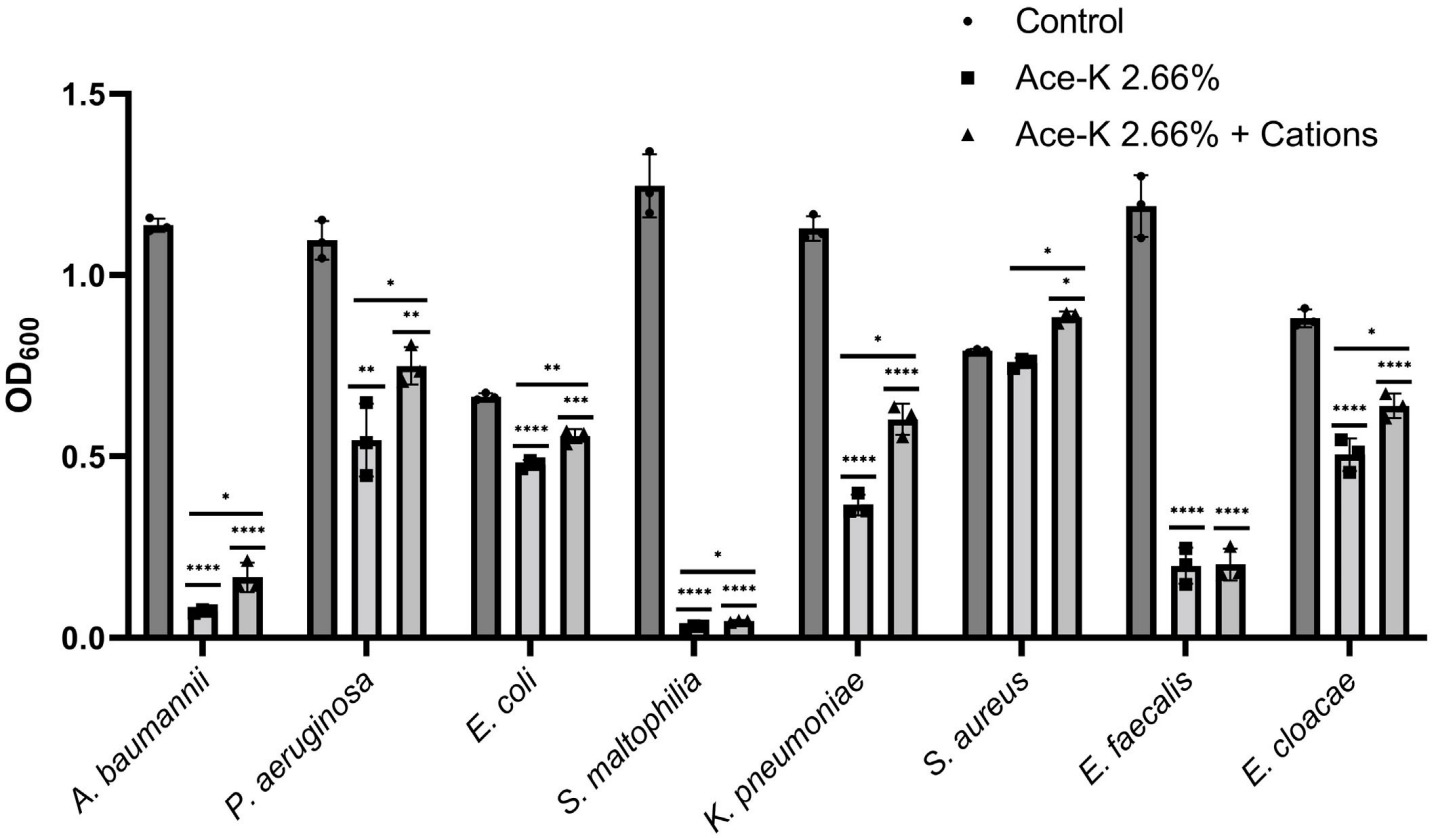
Growth inhibitory activity of 2.66% ace‐K on different clinically relevant bacteria Ace‐K showed a significant inhibitory activity on *A. baumannii* AB5075, *P. aeruginosa* G4R7, *E. coli* NCTC 13476, *S. maltophilia* CCUG 63145, *K. pneumoniae* ST234, *E. faecalis* CCUG 19916T and *E. cloacae* DUB that could be partially complemented, to different extents, by the addition of cations (2 mM CaCl_2_ and 1 mM MgSO_4_). Only S. aureus CCUG 62707 did not show a significant impact on growth when cultured in presence of ace‐K. Bars represent OD_600_ after 24 h. Average of three biological replicates ± S.D. is represented. Analysis was by independent *t*‐test (*P ≤ 0.05, **P ≤ 0.01, ***P ≤ 0.001, ****P ≤ 0.0001).

Given that ace‐K had such a pronounced impact on the growth of *P. aeruginosa* PA14 and *A. baumannii* AB5075, we hypothesised that it may have a similar effect against other clinically relevant pathogens. To explore this, we conducted growth assays in the presence of 2.66% using a set of clinical isolates belonging to a variety of species. Remarkably, this assay demonstrated that ace‐K could significantly inhibit the growth of a number of clinical isolates, including *P. aeruginosa*, *E. coli*, *Stenotrophomonas maltophilia*, *K. pneumoniae*, *E. faecalis* and *E. cloacae* to different extents, depending on the species (Fig 5) but not *Staphylococcus aureus*. The finding that both Gram‐negative and Gram‐positive bacteria could be impacted by ace‐K highlights the potency of its activity.

According to the transcriptomic data, the expression of multiple genes encoding proteins that localise within the cell envelope or that are involved in membrane‐related processes is affected in the presence of ace‐K. This is coherent with previous studies (Yu *et al*, 2021, 2022) showing that wastewater concentrations of ace‐K alter membrane permeability in environmental bacteria. Under membrane stress, bacteria are known to have their growth rate impacted as the cell attempts to balance membrane repair and toxicity (Mitchell & Silhavy, 2019). Furthermore, divalent cations are known to help maintain membrane stability (Clifton *et al*, 2015). To explore the possibility that membrane stability was involved in ace‐K toxicity, we measured the growth of AB5075 in the presence of 2.66% ace‐K compared with a mock treatment and to the same medium supplemented with divalent cations. The addition of cations to *A. baumannii* AB5075 led to a significant increase in growth in the presence of ace‐K (Fig 5). Furthermore, the supplementation with cations was able to rescue the growth of *P. aeruginosa*, *E. coli*, *S. maltophilia*, *K. pneumoniae* and *E. cloacae* in the presence of ace‐K (Fig 5). This suggests that the ace‐K mediated effect on membrane stability is a wide‐spread feature among different pathogenic species. This supports the hypothesis that membrane disruption is, at least, partially responsible for the ace‐K effect on bacterial growth.

### Ace‐K impacts bacterial membrane stability leading to increased permeability, gross morphological distortions and membrane bulging

**Figure 6:**
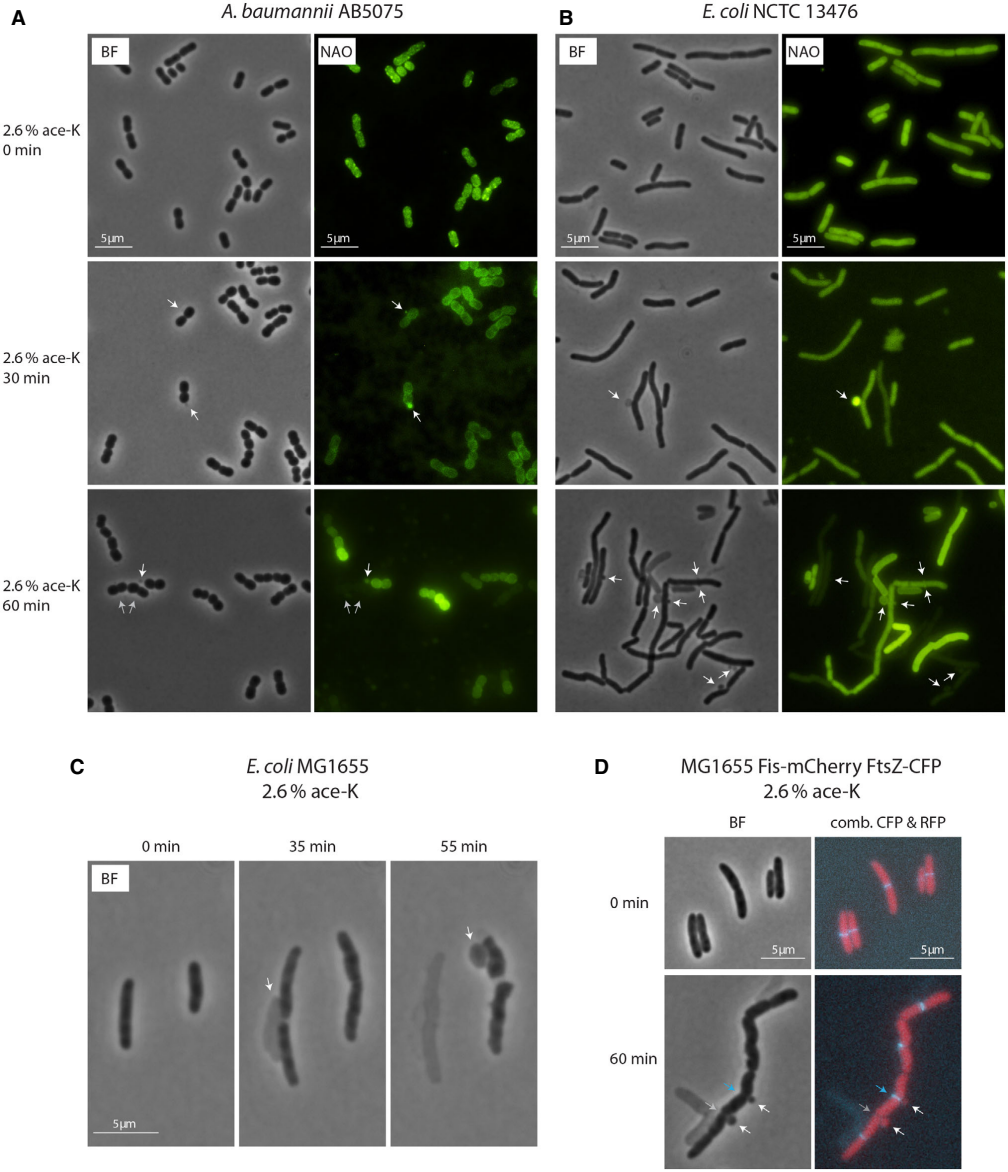
Impact of Ace‐K on cell morphology Phase contrast and fluorescent images of *A. baumannii* cells following treatment with ace‐K. Cells were grown in LB broth to the early exponential growth phase. 2.66% ace‐K was added to the culture and samples were withdrawn at the times indicated. The membrane was visualised by staining with NAO for 5 min before visualisation. Clear examples of membrane bulges are highlighted by white arrows. In later samples an increasing fraction of cells could not be visualised in the fluorescence channel, indicating that they are lysed, leaving only a “ghost” behind, as highlighted in the 60 min figure by two grey arrows.Phase contrast and fluorescent images of the carbapenem‐resistant *E. coli* NCTC 13476. Cells were treated and visualised as described above. White arrows are used to highlight membrane bulges.Phase contrast images of a time lapse of *E. coli* MG1655 cells grown on medium containing ace‐K. An environmental chamber was used to maintain a constant temperature of 37°C and cells imaged for 120 min (see Movies EV3 and EV4). The images shown highlight the extensive bulging that occurs as cells grow in the presence of ace‐K.Phase contrast and fluorescence images of *E. coli* MG1655 cells in which Fis protein are labelled with mCherry and FtsZ protein is labelled with CFP. Cells were treated and visualised as described in (A). The image shows that membrane bulges are clearly visible after 60 min of treatment with ace‐K (white arrows) and occur either at sites where the Z ring is situated (blue arrow) or where invagination has already taken place (grey arrow). Shown are phase contrast images and overlays of the CFP and RFP channels (see Material and Methods for further details).

As mentioned above, our transcriptomic data suggested a disruption of membrane homeostasis. This is reflected in the upregulation and downregulation of 44 and 36 membrane‐related coding genes, respectively (a list of these genes based on their cognate gene ontology terms is provided in Dataset EV3). These rearrangements in the membrane proteome suggest there is a disruption of the membrane homeostasis when cells are treated with this AS. To further explore the impacts of ace‐K on the bacterial cell membrane, we first assessed its effect on membrane permeability using the membrane‐specific dye Nile Red and the DNA stain DAPI (Banerjee *et al*, 2021). This assay confirmed that, when grown in the presence of a sub‐MIC of ace‐K, *A. baumannii* AB5075 showed significant increases in DNA dye uptake as compared to the untreated control (Appendix Fig S8), suggesting a more permeable membrane. It was also noted in this assay that cells exposed to ace‐K appeared to have an altered cell morphology. This observation prompted further investigations into the influence of ace‐K on cell morphology using live cell imaging. By monitoring cell behaviour over time, it was observed that *A. baumannii* cells stop dividing and lose structural integrity, swelling in size rapidly, upon ace‐K exposure. We also observed the formation of bulges in the bacterial cell. Using the Cardiolipin (CL)‐specific fluorescent dye 10‐N‐nonyl‐acridine orange (NAO) to visualise CL distribution (Mileykovskaya & Dowhan, 2000), we could see clear structural rearrangements in the phospholipid composition of the cell membrane and we could also confirm that the bulges were evaginating from cells (Fig 6A; Movies EV1 and EV2). We next sought to explore whether this gross impact on cell morphology was maintained in other species. We repeated our live cell imaging using the carbapenem‐resistant *E. coli* NCTC 13476. Intriguingly, we observed a conserved loss of morphology but distinct from that seen in *A. baumannii*, in that instead of cells swelling, *E. coli* cells filamented, extending to many times their original size before eventually forming characteristic membrane bulges and ultimately lysing (Fig 6B). These phenotypes were also conserved in the lab *E. coli* strain MG1655 (Fig 6C; Movies [Link], [Link]). To better understand the localisation of these membrane bulges and to gain insight into the contents of the bulges, we performed live imaging microscopy of an *E. coli* MG1655 strain with labelled mCherry‐Fis and CFP‐FtsZ. Fis is a small DNA‐binding protein that binds to a large number of regions of the chromosome (Cho *et al*, 2008), allowing the visualisation of the nucleosome in living cells. FtsZ is a component of the Z ring (Bi & Lutkenhaus, 1991), showing future cell division sites. This time‐lapse experiment revealed that the membrane bulges were largely localised to either a site where a septum is formed or at a site where invagination has already taken place. The mCherry‐Fis also showed that these bulges contain nuclear material (Fig 6D). This suggests that the mechanism by which ace‐K is leading to cell death is through bulge‐mediated cell lysis.

### Ace‐K resensitises MDR *A. baumannii* against different antibiotics

**Figure 7:**
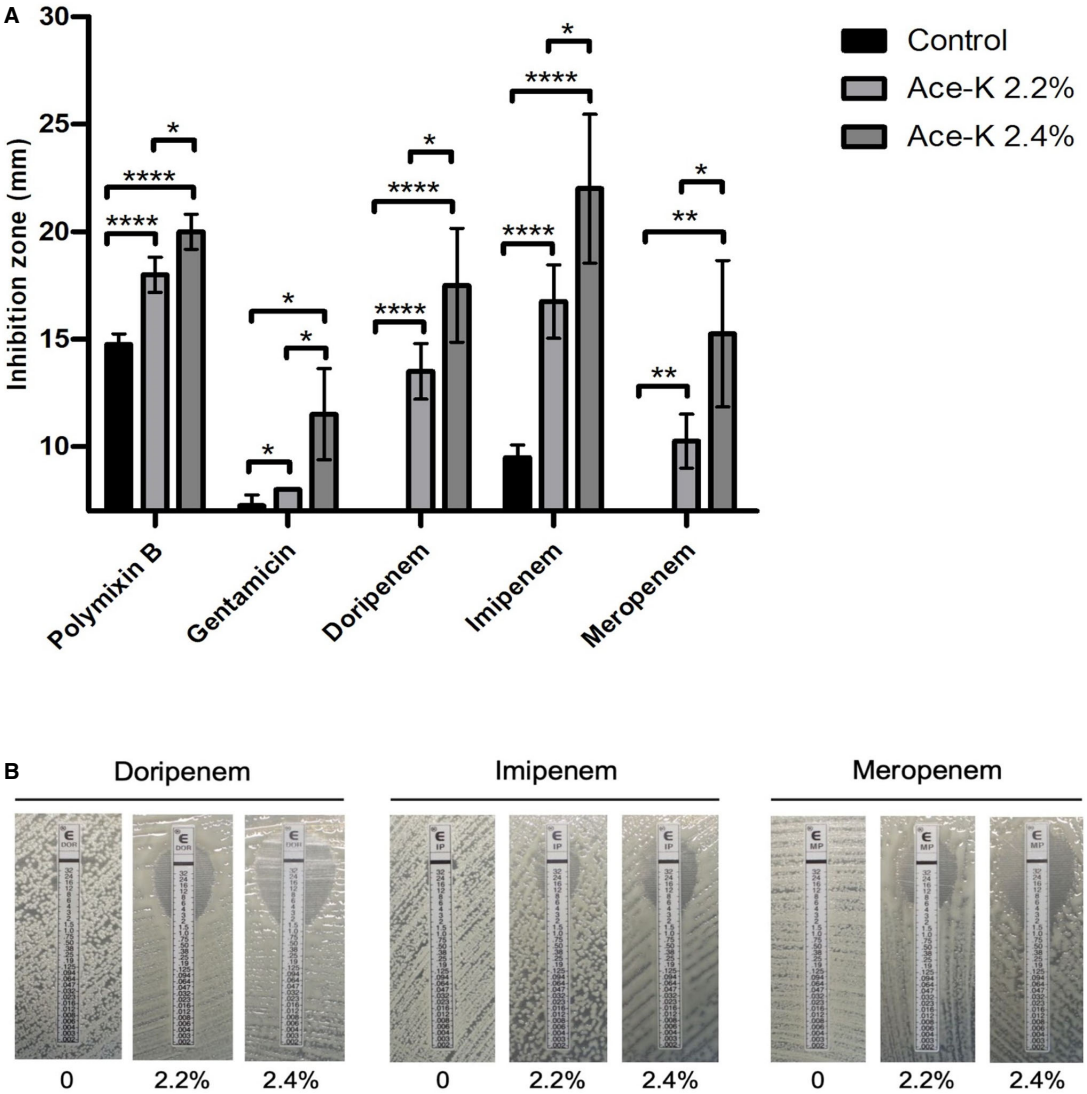
Ace‐K increases the sensitivity of *Acinetobacter baumannii* AB5075 to different antibiotics A, BA dose‐dependent effect was observed correlating the concentration of ace‐K with the sensitivity of AB5075 to polymyxin B, gentamicin and the carbapenems doripenem, imipenem and meropenem (A). Giving the trend on the specific effect on carbapenems, an Etest MIC assay was conducted (B), further demonstrating the increased sensitivity in the presence of ace‐K. The average of three biological replicates and representative photographs out of four biological replicates ± S.D. Analysis was by independent *t*‐test (*P ≤ 0.05, **P ≤ 0.01, ****P ≤ 0.0001).

*A. baumannii* is a notoriously difficult pathogen to treat and is recognised for its remarkable ability to evolve resistance to almost all available antibiotics (Harding *et al*, 2018; Tacconelli *et al*, 2018). This is due to the joint contribution of its cell envelope acting as a barrier and the considerable repertoire of detoxifying enzymes and efflux pumps it encodes in its chromosome and its accessory genome (i.e. plasmids and mobile genetic elements; McCarthy *et al*, 2021). Because of the role of the cell envelope in antibiotic resistance, and since ace‐K produces membrane alterations and affects its stability, we considered the hypothesis that it may render bacteria more susceptible to antibiotic treatment. To explore this, we tested the susceptibility of *A. baumannii* AB5075 to a panel of different antibiotics in the presence of ace‐K concentrations that would impact growth without completely inhibiting it (2.2 and 2.4%). According to the results (Fig 7A), ace‐K potentiates the activity of polymyxin B, gentamicin and the carbapenems doripenem, imipenem and meropenem. This strain is known to be highly resistant to carbapenems. Given this striking effect on carbapenem resistance, we quantified the MIC change for these antibiotics in the presence of ace‐K (Fig 7B; Table EV1). In the absence of ace‐K the carbapenem MIC was above the detection limit of the test (32 mg/l), a concentration of 2.4% ace‐K dropped these MICs to 1.9, 4.5 and 1.8 mg/l for doripenem, imipenem and meropenem, respectively. We further tried to extrapolate this resensitisation to other β‐lactams by performing disc diffusion assays. However, we would only observe similar changes in susceptibility using discs loaded with 40 to 100‐fold higher concentrations compared to those of carbapenems (Appendix Fig S9). Considering its inhibitory, anti‐virulence and antibiotic potentiator effect against multidrug‐resistant pathogens, ace‐K may stand as a promising candidate in combination therapies for infection treatment.

### Ace‐K loaded wound washes and dressings decrease bacterial viability in colony biofilms *in vitro*

**Figure 8:**
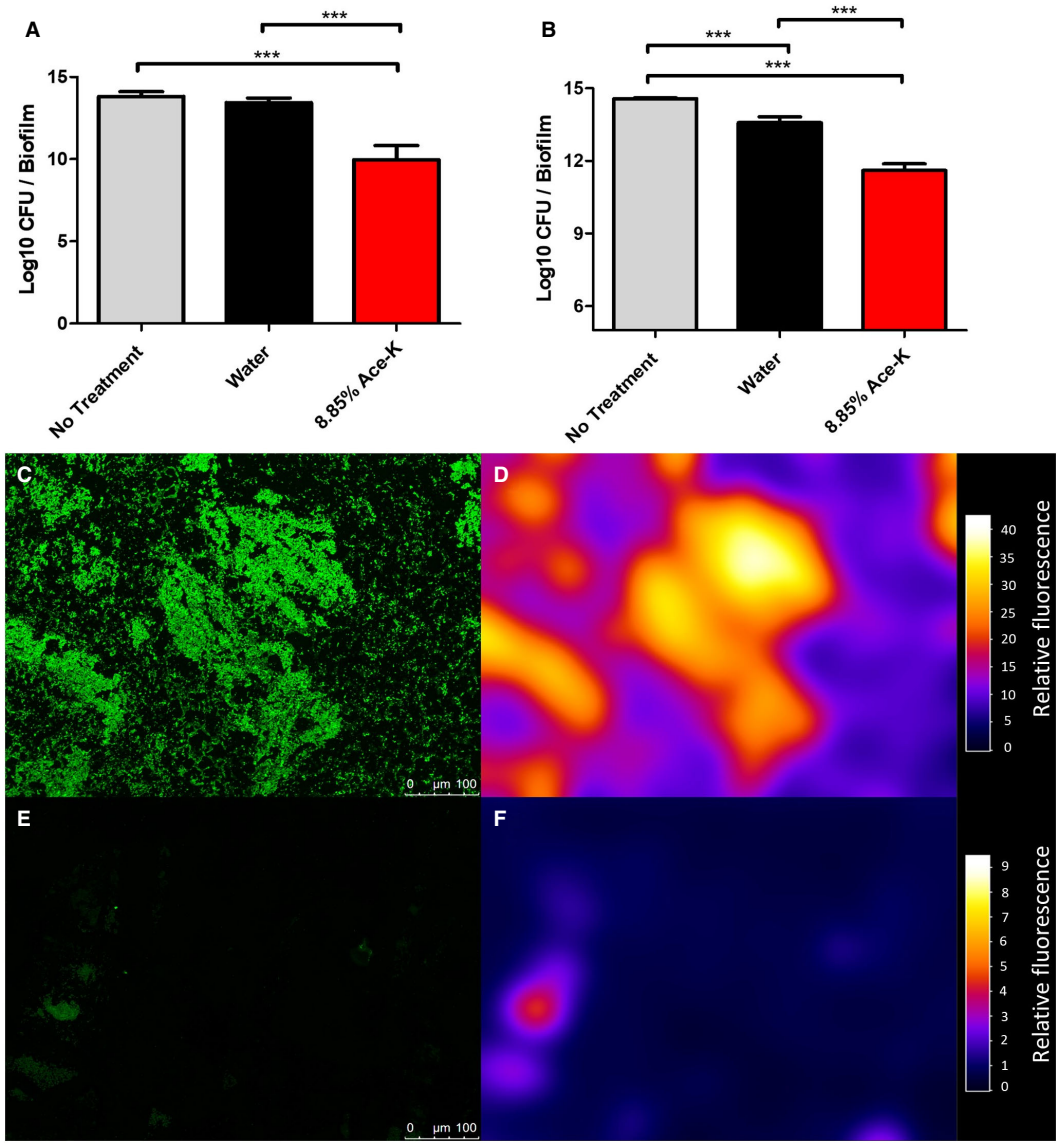
Ace‐K and colony biofilms A–FAntimicrobial activity of 8.85% ace‐K against *A. baumannii* AB5075 colony biofilms when applied as a (A) wash solution and (B) a soaked gauze, (C, E) fluorescence images and (D, F) fluorescence intensity mapping of ace‐K gauze treated biofilm. Washing colony biofilms with ace‐K solution (A) resulted in a 3.47 log reduction in the number of viable, biofilm‐bound cells. When applied as a saturated gauze (B), ace‐K treatment yielded a 2‐log reduction in viable cells compared with the water control. Average of three biological replicates ± S.D. are represented (***P ≤ 0.001). Analysis was by independent *t*‐test. *A. baumannii* biofilm grown on glass microscope slides (C, D) showed a substantial reduction in viable biofilm‐bound cells when treated for 1 h with ace‐K gauze (E, F). Mapping fluorescence intensity of the image showed significant reductions in fluorescence, indicative of a reduction in SYTO9‐stained cells. A three‐dimensional visualisation of fluorescence intensity maps is shown in Appendix Fig S10.

Bacterial pathogens can form biofilms in infected wounds. These biofilms are highly resistant to treatment and lock the wound in persistent inflammatory state, leading to the development of a chronic wound (Maslova *et al*, 2021). The antimicrobial activity of ace‐K holds significant clinical potential particularly as AS are generally regarded as safe by regulatory administrations (WHO Food Additives Series 28). Considering its anti‐virulence properties, we hypothesised that ace‐K could be applied directly on an established biofilm to disrupt it. To initially assess this, *A. baumannii* AB5075 colony biofilms were grown and subsequently exposed to a wash with an 8.85% ace‐K solution, simulating a wash/irrigation of a chronically infected wound (Giri *et al*, 2020; Lewis & Pay, 2022). This treatment, when compared to an untreated biofilm and to a water wash control, led to a 3.5 log reduction in the number of viable cells within an AB5075 biofilm (Fig 8A). In order to know whether ace‐K could have a similar impact on chronic wound colonisation when carried in an augmented wound dressing, we performed a similar colony biofilm assay but covered the biofilm in a surgical gauze soaked in an 8.85% ace‐K solution. After 1 h of treatment, the AB5075 biofilm showed a 1.98 log reduction in viable cells compared with the water‐soaked gauze control (Fig 8B). Further, we showed that following treatment with ace‐K soaked gauze, 18‐h old AB5075 biofilms grown on glass slides had a significant reduction in overall biomass, as indicated by a reduction in total fluorescence intensity (Fig 8C–F). Together, these results clearly demonstrate the potential of ace‐K in wound treatments.

### Ace‐k‐loaded wound dressing decreases bacterial viability in burns and acute lacerations in an *ex vivo* porcine skin model

**Figure 9:**
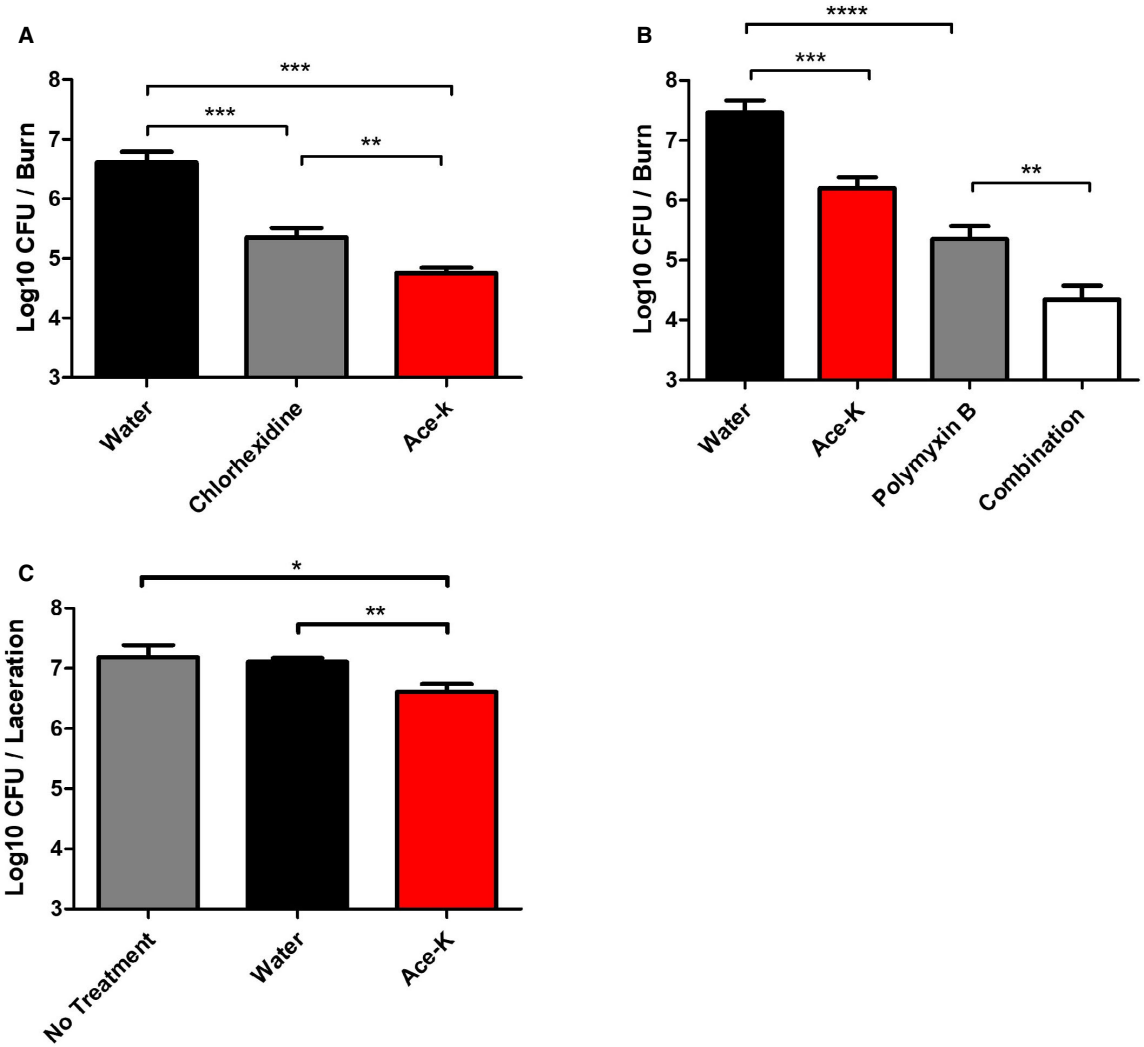
Therapeutic potential in an *ex vivo* model A–CAssessment of ace‐K as an individual treatment in comparison with current wound disinfectant, as an adjuvant treatment with polymyxin, and as treatment for infected lacerations using an *ex vivo* porcine skin model. Assessment of a gauze soaked in an 8.85% ace‐K solution as a stand‐alone treatment of an infected burn (A) showed a 1.86 log reduction in viable biofilm‐bound cells compared with a vehicle control while the commonly used wound disinfectant Sterisept yielded only a 1.27 log reduction. When used in conjunction with polymyxin (B), ace‐K increased the efficacy of the antibiotic from a 2.11 log reduction when used alone, to a 3.13 log reduction when used with ace‐K. When used to treat an infected laceration (C) a 0.5 log reduction was achieved. Data shown represent the average of three biological replicates ± S.D. Analysis was by independent *t*‐test (*P ≤ 0.05, **P ≤ 0.01, ***P ≤ 0.001, ****P ≤ 0.0001).

The response of antibiotic‐resistant organisms to antimicrobial treatment is significantly affected by the organism's microenvironment and nutrient availability (Maslova *et al*, 2021; Van den Bossche *et al*, 2021). To assess the efficacy of ace‐K against *A. baumannii* within a wound infection environment, we used an *ex vivo* porcine wound infection model (Alves *et al*, 2018). Biofilms were grown in either a burn wound or a skin laceration on porcine skin explants and treated with an ace‐K soaked gauze dressing. To assess ace‐K against a currently used treatment, we used Sterets Unisept, which contains chlorhexidine as the active ingredient. This was selected as a comparator treatment, as clinicians avoid the use of topical antimicrobials due to unclear efficacy and difficulty in achieving an accurate dose (Lipsky & Hoey, 2009). After 1 h of treatment with ace‐K alone, the AB5075 biofilm grown in the burn model showed a 1.86 log reduction in viable cells compared with the water‐loaded gauze control treatment (Fig 9A), whereas biofilms treated with Sterisept showed only a 1.27 log reduction.

To assess the potential of ace‐k as part of a combination therapy, burn wound biofilms were treated with either a gauze loaded with 1.5 ml of 0.59 mg/ml polymyxin B (a dose equivalent to the commonly used, polymyxin‐containing topical cream Neosporin). Burn wound biofilms treated with polymyxin B showed a 2.11 log reduction in viable cells while treatment with a combination of polymyxin B and ace‐K showed an improved log reduction of 3.13 (Fig 9B). Finally, we assessed ace‐K soaked gauze as a potential treatment of infected laceration wounds. When infected laceration wounds were treated with 8.85% ace‐K a 0.5 log reduction in viable cells was achieved over the water control (Fig 9C). These results reinforce the potential of ace‐K as a treatment in infected wounds in a model that is more comparable to a clinical scenario.

## Discussion

The western diet is filled with compounds that alter bacterial behaviour. Many of these compounds have been shown to disrupt bacterial communications systems, such as quorum sensing, and significantly alter the composition of the gut and oral microbiome (McCarthy & O'Gara, 2015). Findings like these have prompted further research exploring the capacity of these compounds to influence infection progression. Garlic oil, for example, is known to contain a range of compounds capable of inhibiting quorum sensing in *P. aeruginosa* and has shown promising results in clinical trials when administered to cystic fibrosis patients with chronic *P. aeruginosa* lung infections (Smyth *et al*, 2010). Cinnamaldehyde, found in cinnamon, has also been shown to inhibit quorum sensing in this pathogen (Ahmed *et al*, 2019). However, the range of compounds found in the diet goes beyond those of natural origin. Since their introduction in the food industry, AS have caught the attention of the scientific community, mainly regarding their effect on human health. More recently a focus has been placed on how AS influence the microbiome. The variety of studies focused on this phenomenon agreed that AS can alter the behaviour of the microbial communities in the gut, showing that these compounds indeed exhibit a biological activity.

In this work, we tackle, for the first time, the effect of AS on two relevant pathogenic bacteria: *A. baumannii* AB5075 and *P. aeruginosa* PA14. We assessed the impact of a panel of AS on the growth of these organisms (Figs 1 and 2; Appendix Figs S1–S3), obtaining results ranging from growth enhancing to different degrees of growth inhibition depending on the pathogen. However, ace‐K showed a strong effect on both *A. baumannii* and *P. aeruginosa*. We, therefore, focused on this AS for further experimental development. We demonstrate that ace‐K has potent anti‐biofilm activity at sub‐inhibitory concentrations and also has the capacity to disrupt established biofilms. This anti‐biofilm effect appears to be mediated through the upregulation of the whole *paa* phenylacetic acid degradation pathway (ABUW_2525–2536). This pathway has been recently shown to significantly influence the expression of the *csu* operon and therefore biofilm levels (Moon *et al*, 2017; Hooppaw *et al*, 2022). In later experiments, we observed that ace‐K has a broad range of antimicrobial activity (Fig 5), ranging from a complete growth inhibition of *S. maltophilia* to no impact in *S. aureus*. The lack of an effect on the growth of *S. aureus* suggests this bacterium has the means of overcoming the toxic effects of ace‐K exposure, which certainly warrants further investigation.

We conducted a dRNA‐seq experiment to reveal the transcriptional alterations caused by ace‐K in *A. baumannii* AB5075. Previous global transcription examinations have shown that ace‐K, at concentrations in which it is found in wastewater (around 10^7^‐fold below our working concentrations) induced changes in *E. coli* and *A. baylyi* transcriptomes (Yu *et al*, 2021, 2022). However, those effects varied between species. For example, ace‐K triggered reactive oxygen species (ROS) production in *E. coli*, whereas the same range of concentrations did not increase ROS in *A. baylyi*. The fact that, even at these low concentrations, ace‐K produced distinct responses in two bacterial species supports the previous hypothesis that different species will react in different manners to ace‐K. Interestingly, transcriptomic and phenotypic studies on the naturally competent *A. baylyi* (Yu *et al*, 2022) showed that ace‐K induced the expression of *pil* gene (encoding T4P; Ellison *et al*, 2021; Vesel & Blokesch, 2021) and promoted natural transformation. Conversely, at our working ace‐K concentrations, we have shown a significant downregulation of those gene clusters at higher concentrations, resulting in a nonmotile phenotype and the abolition of natural transformation in *A. baumannii*. This clearly indicates that the effect of ace‐K on bacteria varies in a dose‐dependent manner.

dRNA‐seq analysis also uncovered that a large proportion of the genes that were differentially regulated encoded proteins associated with the membrane (Dataset EV3). This suggested that ace‐K is having an influence on cell membrane integrity. When assessed, a clear impact in membrane permeability was seen by increased DNA dye uptake (Appendix Fig S8). Live cell imaging revealed the extent to which cell morphology is impacted due to ace‐K exposure in *E. coli* and *A. baumannii*, with cells filamenting or ballooning, respectively. As cells lost their native morphology, clear membrane bulges were seen to appear, followed by cell lysis/death. This mechanism of cell death, where cells lose morphological integrity and bulges form in the membrane prior to lysis, is reminiscent of the specific morphological effects of β‐lactam antibiotics on the cell (Chung *et al*, 2009; Wood *et al*, 2019). Indeed, it has been shown previously that the addition of cations can stabilise these β‐lactam induced bulges allowing cell survival (Yao *et al*, 2012). Given that we have already demonstrated that cells can be partially rescued from the effects of ace‐K through cation supplementation and the visual evidence of membrane bulges forming in different species when exposed to ace‐K, then it strongly supports that the antimicrobial effect of ace‐K is through bulge‐mediated cell lysis. This effect may be mediated at the level of the membrane or, given that there are peptidoglycan amide and glycoside hydrolases that are differentially expressed in the dRNA‐seq dataset (e.g. ABUW_0928, ABUW_3204, ABUW_4116, ABUW_0928 and ABUW_4116), it may be through a disturbance in the balance of peptidoglycan growth and degradation dynamics and turnover of the aberrant cell wall (Dik *et al*, 2017), leading to a loss of the cell envelope structural integrity. Furthermore, this phenomenon might also be involved in the increased susceptibility to carbapenems in the presence of ace‐K.

A combination of features in *A. baumannii* confers a robust multidrug resistance profile that makes this organism recalcitrant to antibiotic treatment (McCarthy *et al*, 2021). For this reason, finding alternatives to disrupt these resistance mechanisms has become a major task for future therapies. We have shown that sub‐lethal concentrations of ace‐K have the ability to increase the sensitivity of *A. baumannii* to a number of antibiotics, particularly to carbapenems. The first barrier that carbapenems encounter is the outer membrane, which they need to penetrate in order to reach the periplasmic space, where they develop their activity. We have shown that membrane permeability increased and that cells undergo gross morphological changes (Fig 6; Appendix Fig S8). Together, these data suggest that ace‐K is increasing the carbapenem penetration rate by permeabilising the outer membrane.

The growth inhibition and anti‐virulence effects displayed by the AS tested in this study suggest that they may have therapeutic potential to prevent or treat infections. To test this, we used an *in vitro* colony biofilm model and two different applications of ace‐K: a wash and a soaked gauze. A wash was chosen as it would facilitate greater penetration of the sweetener into an established biofilm (Lewis & Pay, 2022), and a soaked gauze, as it could enable prolonged localised concentrations of ace‐K. Both applications led to a significant reduction in viable bacterial numbers. To further test the clinical potential of AS, we assessed the capacity of ace‐K to treat infected burn and physical trauma wounds in the porcine *ex vivo* skin model (Alves *et al*, 2018). Wound care and management is a huge economic burden on health care systems, with the UK's National Health Service (NHS) spending an estimated £8.3 billion pounds annually on wound management (Guest *et al*, 2020). Infection is one of the main clinical complications associated with wound care and, as such, oral antibiotics are regularly prescribed prophylactically to minimise the risk of bacterial colonisation, particularly in surgical wounds. The application of ace‐K as an augmented gauze dressing led to a significant reduction of viable bacteria in the burn and laceration models with only a single 1‐h treatment. Indeed, this formulation led to a greater reduction in viable bacteria than a chlorhexidine‐based wound antiseptic in a porcine *ex vivo* wound model (Fig 9B). The topical application of antibiotics is relatively rare due to difficulties with accurate dosing; however, polymyxins have been used relatively routinely in topical treatments, as they limit the neurotoxicity and nephrotoxicity associated with systemic polymyxin administration (Shatri & Tadi, 2022). Ace‐K showed an ability to potentiate the activity of polymyxin B *in vitro* and when assessed in the *ex vivo* wound model, a significant decrease in viable bacteria was seen in the combination treatment, as compared to either application alone. These findings suggest it could provide a robust option, either singularly or in combination, to treat infections associated with pathogens that are resistant to frontline therapy.

A particular advantage to the further preclinical development of AS, such as ace‐K, as therapeutics or adjuvants, is that they are FDA approved and, as such, there have been numerous studies into their safety even at concentrations that far exceed the recommended daily intake of 15 mg/kg. For example, mouse trials in which animals were fed up to 5,700 mg/kg a day for 40 weeks and showed no ill effects and there was no evidence of inflammation in any tissue as a result of this exposure (Sinkeldam *et al*, 1991; National Toxicology Program, 2005; Chappell *et al*, 2020). Moreover, ace‐K has also shown no impact on the immune system (Reuzel & van der Heijden, 1991). Given the proliferation of this and other AS in the diet and the lack of regulation with respect to levels of consumption at an individual level, the likelihood is that these compounds reach much higher concentrations than the recommended daily intake in the body, particularly in the mouth and gut. However, further work is needed to fully explore the clinical potential of AS, particularly in a wound setting. Nevertheless, this work establishes the foundations for the future development of therapies based on AS as wound treatments, and even as antibiotic adjuvants, given their ability to resensitise pathogens to already existing antimicrobials.

## Materials and Methods

### Bacterial strains and growth conditions

*A. baumannii* AB5075 (colony type VIR‐O; Chin *et al*, 2018), *P. aeruginosa* PA14 and the rest of the bacterial species used in this work were routinely grown in liquid (shaking 180 rpm) or solid LB medium (Miller), unless otherwise stated, at 37°C. When necessary, LB was supplemented with CaCl_2_ 2 mM and MgSO_4_ 1 mM. Bacterial strains, plasmids and oligonucleotides used in this work are listed in Table EV2. All plasmids were stored in *E. coli* DH5⍺ or DH5⍺ λ*pir*, according to their origin of replication.

### Plasmid and strain construction

A pUC18T‐miniTn7T‐lacI^q^‐Ptac derivative was constructed to produce selectable miniTn7T insertions in AB5075. For that, the tetracycline resistance gene *tetA* was amplified from pSEVA524 (Silva‐Rocha *et al*, 2013) using oligonucleotides tetA fw and tetA rv (de Dios *et al*, 2022). The PCR product was ligated into pUC18T‐miniTn7T‐Gm‐lacI^q^‐Ptac (Meisner & Goldberg, 2016; Addgene #110558) digested with EagI and BsrGI and blunted with Klenow, generating pUC18T‐miniTn7T‐Tc‐lacI^q^‐Ptac.

A miniTn7‐based *PpilA::gfpmut3* transcriptional fusion was constructed for monitoring the expression from the *PpilA* promoter. A *PpilA* PCR product was amplified using oligonucleotides PpilA fw EcoRI and PpilA rv BamHI. Then, it was digested and ligated into pUC18T‐miniTn7T‐zeo‐gfpmut3 (Choi & Schweizer, 2006; Addgene #65037), resulting in pUC18T‐miniTn7T‐zeo‐PpilA::gfpmut3.

All cloned fragments were validated by Sanger sequencing.

*A. baumannii* AB5075 miniTn7T insertions (AB5075/miniTn7T‐Tc‐lacI^q^‐Ptac, AB5075/miniTn7T‐zeo‐gfpmut3 and AB5075/miniTn7T‐zeo‐PpilA::gfpmut3) were generated by four‐parental mating as previously described (Ducas‐Mowchun *et al*, 2019) using pRK2013 and pTNS2 as helper plasmids (Figurski & Helinski, 1979; Choi *et al*, 2005). Clones bearing the respective insertions were selected in tetracycline (5 mg/l) or zeocin (250 mg/l), according to the resistance marker borne in the miniTn7T backbone, and validated by PCR using primers AB5075‐glmS fw and Tn7R (Kumar *et al*, 2010).

### MIC and MBIC determination

Overnight cultures were diluted in 96‐well plates to OD_600_ 0.1 for *P. aeruginosa* PA14 and 0.05 for *A. baumannii* AB5075 in LB medium supplemented with ace‐K in concentrations ranging from 0.09 to 7.08%, including a vehicle control for each ace‐K dilution. Cultures were incubated at 37°C and 180 rpm for 18 h. Following incubation, planktonic growth was assessed by OD_600_ reading. The MIC was defined as the lowest concentration of ace‐K, which resulted in a significant decrease in planktonic growth compared with the corresponding vehicle control.

Following measurements of planktonic growth, biofilm formation was measured. Media and planktonic cells were removed from the wells and biofilms were gently washed with deionised water three times. Then, 200 μl of 0.1% crystal violet was added to each well and plates were incubated statically for 15 min at room temperature. The stain was then removed and wells were washed five times with deionised water. After leaving the plates to air‐dry, the retained crystal violet was re‐solubilised by adding 200 μl of 99% ethanol to each well and incubating statically at room temperature for 6 h. Crystal violet was quantified by measuring absorbance at 570 nm. The MBIC was defined as the lowest concentration of ace‐K, which resulted in a significant decrease in biofilm formation compared with the corresponding vehicle control. MIC and MBIC results represent averages of three independent replicates (three technical replicates per experiment).

### Biofilm eradication assay

To assess the ability of ace‐K to eradicate established biofilms, overnight cultures were diluted in 96‐well plates to OD_600_ 0.1 for *P. aeruginosa* PA14 and 0.05 for *A. baumannii* AB5075 in the LB medium. Plates were incubated for 18 h at 37°C and 180 rpm to allow biofilms to form. Following incubation growth medium was removed from the wells and biofilms were washed three times with 200 μl of sterile PBS to remove any unbound planktonic cells. Fresh LB medium supplemented with 8.85% ace‐K or an appropriate vehicle control was added to the wells. Plates were incubated for a further 24 h at 37°C and 180 rpm. Following this treatment biofilms were stained with 0.1% crystal violet as detailed above. Reduction in biofilm was represented as a percentage reduction compared with the control.

### dRNA‐seq and gene enrichment analysis

Cells were grown to mid‐exponential phase (OD_600_ 0.6–0.7) in 20 ml LB supplemented with either 1.3% ace‐K or the matching volume of vehicle control. Cells were spun down and washed in RNAlater to preserve RNA integrity. RNA was isolated using the RNAeasy Kit with on‐column DNAase digestion (Qiagen). RNA integrity was determined using a Bioanalyzer. Samples were further processed for RNA sequencing on an Illumina MiSeq with 12 million reads per sample. Sequencing and downstream analyses were performed at Microbial Genome Sequencing Center (Pittsburgh, Pennsylvania, U.S.A.). Quality control and adapter trimming was performed with bcl2fastq. Read mapping were performed with HISAT. Differential expression analysis was performed using the edgeR's exact test for differences between two groups of negative‐binomial counts with an estimated dispersion value of 0.1. and using the *A. baumannii* AB5075‐UW genome annotation as reference (Gallagher *et al*, 2015). 464 genes were identified as being significantly differentially expressed greater than ¦logFC¦ > 1 and *P* < 0.05. Significantly regulated genes were subjected to Gene Set Enrichment Analysis (FUNAGE‐Pro v1.0) for functional clustering (de Jong *et al*, 2022).

### GFP‐based gene expression assays

The expression from the *PpilA* promoter was measured using a miniTn7T‐based insertion bearing a *PpilA::gfpmu* transcriptional fusion (AB5075/miniTn7T‐zeo‐PpilA::gfpmut3). An AB5075/miniTn7T‐zeo‐gfpmut3 strain, bearing the empty miniTn7T backbone, was used as a control. Overnight cultures of strains bearing either the *PpilA::gfpmut3* fusion or the empty transposon were diluted 1:100 (v/v) in fresh LB broth supplemented with 0.33, 0.66 or 1.33% ace‐K, or a mock treatment. Cultures were incubated for 2 h at 37°C, 180 rpm. Then, samples were withdrawn from the cultures, washed with PBS and eventually resuspended in PBS. Two technical repeats of each sample were allocated in a 96‐well plate and their OD_600_ and GFP fluorescence (excitation: 485 nm; emission: 535 nm) were measured. The fluorescence readings were normalised by their respective OD_600_ and the baseline fluorescence obtained from the empty transposon control was subtracted from that obtained with the strain bearing the *PpilA::gfpmut3* the promoter fusion measurements. Three biological replicates were performed for each experimental condition.

### Twitching motility assays

Twitching assays were performed in twitching media (tryptone 10 g/l, yeast extract 5 g/l, NaCl 2.5 g/l, agar 10 g/l). After autoclaving, differing amounts of ace‐K or water (adjusted to add the same volume) was added to the media, mixed thoroughly and 10 ml of solution poured into 90 mm Petri dishes. The open plates were allowed to cool for 10 min next to a Bunsen burner. To inoculate plates, a colony was picked from a freshly grown plate culture with a pipette tip and stabbed to the bottom of the plate. Plates were incubated at 37°C for 48 h. Five biological replicates were performed for each experimental condition.

### Natural transformation assay

Natural transformation assays were performed following the protocol published by Vesel & Blokesch (2021) with modifications. Stationary phase *A. baumannii* AB5075 cultures were diluted 1:100 (v/v) in fresh LB medium supplemented with differing amounts of ace‐K or a water control. When necessary, the medium was also supplemented with CaCl_2_ 2 mM and MgSO_4_ 1 mM. Cultures were incubated at 37°C, 180 rpm until reaching OD_600_ 0.5. Next, 20 μl of cell culture were mixed with 800 ng of purified genomic DNA from a wild type AB5075 bearing a site‐specific miniTn7T‐Tc derivative transposon (AB5075/miniTn7T‐Tc‐lacI^q^‐Ptac, Tc^r^) inserted in the chromosome (Ducas‐Mowchun *et al*, 2019). The mixtures were patched on LB agar (pH 6.0) carrying the same supplements as previously mentioned and were left to air‐dry in a laminar flow hood, followed by an incubation of 4 h at 37°C. Then, the biomass was resuspended in 1 ml of LB broth and serial dilutions were plated on selective LB agar (tetracycline 5 mg/l) for transformant cells or on plain LB agar to assess viability. Transformation frequency was calculated as the number of transformant cells per millilitre divided by the number of viable cells per millilitre and represented in a log‐scaled axis. Five biological replicates were performed for each experimental condition.

### Differential fluorescence microscopy for the assessment of membrane permeability

Membrane permeability was assessed using two fluorescent stains, Nile Red and DAPI, which stain the bacterial membrane and chromosomal DNA, respectively. Cultures of AB5075 of OD_600_ 0.05 were prepared in 15 ml of either LB broth containing 1.33% ace‐K or an equivalent volume of water in a 100 ml Erlenmeyer flask. Cultures were incubated at 37°C and 180 rpm shaking for 2 h. Following incubation 10 μl of a 1 mg/ml DAPI solution and 10 μl of a 5 mg/ml solution of Nile Red were added to each flask before returning to the incubator for 30 min. Once stained, cultures were centrifuged at 5000 rpm for 5 min and the supernatant discarded. Pellets were resuspended in 10 ml of sterile 4% formaldehyde in PBS and incubated in the dark for 30 min to fix. Once fixed, samples were centrifuged at 5000 rpm and pellets were washed twice with 10 ml sterile PBS. After washing pellets were resuspended in 10 ml of sterile PBS and 10 μl of the cell suspension was spotted onto a glass slide and allowed to air‐dry in the dark. Three spots were prepared per flask. A cover slip was affixed to the slide and samples were imaged using Leica HF14 DM4000 microscope using CY3 (Ex: 542–568 nm, Em: 579–631 nm) and DAPI (Ex 325–375 nm, Em: 435–485 nm) filters. The native Leica Application Suite Advanced Fluoresence software (V4.0.0.11706) was used for image capture. Images were captured at 400x magnification using an exposure of 876 ms for both stains and a gain of 2.5.

### Single‐image microscopy

Fresh overnight cultures of strains of interest were diluted 1:100 (v/v) in fresh LB broth (Miller) and incubated with vigorous aeration at 37°C until OD_600_ reached 0.2. Ace‐K was added at the required concentration and the first sample removed immediately. The following samples were taken at the times indicated. For staining of the membrane, 10‐nonyl acridine orange (Invitrogen™) was added to the sample to a final concentration of 200 nM and incubated for 5 min at room temperature. 1 μl of the sample was pipetted onto an agarose pad and air‐dried. For the generation of pads, a 65 μl (15 × 16 mm) GeneFrame (Thermo Scientific™) was added to a conventional microscopy slide. 1% of SeaKem LE agarose (Lonza) was added to 1 × M9 minimal medium (diluted from a 5 × stock, Sigma‐Aldrich) and heated until the agarose was completely dissolved. 95 μl of the solution was added into the GeneFrame chamber and the chamber sealed immediately with a conventional microscopy slide. Once set, the top slide was removed and the agarose pad air‐dried for 20 min at room temperature and used immediately. Once the sample was added and air‐dried the GeneFrame chamber was sealed by adding a 22 × 22 mm cover slip. Visualisation was done using a Ti‐U inverted microscope (Nikon) with a CFI Plan Fluor DLL 100 × objective (Nikon) and an ORCA Flash 4.0 LT plus camera (Hamamatsu). Phase contrast images were taken using a pE‐100 single LED wavelength source (CoolLED). For fluorescence, the pE‐4000 illumination system (CoolLED) was used. The relevant filters for visualisation of CFP, 10‐nonyl acridine orange and mCherry were Zeiss filter sets 47 (CFP) and 46 (YFP), as well as Nikon TXRED‐A‐Basic Filter (mCherry). Images were captured using the NIS Elements‐BR software V4.51 (Nikon) and exported to tiff. Postprocessing, such as cropping and rotating, was performed in Adobe Photoshop CC (V23.0.0).

### Time‐lapse microscopy

Fresh overnight cultures of strains of interest were diluted 1:100 (v/v) in fresh LB broth (Miller) and incubated with vigorous aeration at 37°C until OD_600_ reached 0.2. 1 μl of the sample was pipetted onto an agarose pad and air‐dried. For the generation of pads, 65 μl (15 × 16 mm) GeneFrames (Thermo Scientific™) were used. Five GeneFrames were stacked on top of each other and added to a conventional microscopy slide. 1% of SeaKem LE agarose (Lonza) was added to LB broth (Miller composition) and heated until the agarose was completely dissolved. If required ace‐K was added to the molten agarose solution at the required concentration. 500 μl of the solution was added into the chamber of the stacked GeneFrames and the chamber sealed immediately with a conventional microscopy slide. Once set, the top slide was removed and 2‐mm‐wide vents were cut into the GeneFrame stack on all four sides. The agarose pad was then air‐dried for 20 min at room temperature and used immediately. Once the sample was added and air‐dried, the GeneFrame chamber was sealed by adding a 22 × 22 mm cover slip. Cells were visualised using the Ti‐U system described above. For time‐lapse imaging, the temperature was maintained at 37°C using an environmental chamber (Digital Pixel). Time‐lapse stacks were captured using the NIS Elements‐BR software V4.51 (Nikon) and either exported to mp4 or tiff. Postprocessing of tiff images, such as cropping and rotating, was performed in Adobe Photoshop CC (V23.0.0).

### Antibiotic sensitivity test

Antibiotic sensitivity assays were performed in cation‐adjusted Mueller‐Hinton pH 7.4 medium (CAMH, Sigma‐Aldrich; CaCl_2_ 2 mM, MgSO_4_ 1 mM). Overnight cultures of *A. baumannii* AB5075 were diluted to 0.5 McFarland units in CAMH broth and spread on CAMH agar plates supplemented with ace‐K 2.2 or 2.4% (or a mock treatment) with a sterile swab. For inhibition zone measurement, polymyxin B (300 U), gentamicin (10 μg), doripenem (10 μg), imipenem (10 μg) or meropenem (10 μg) discs (Oxoid) were placed in the middle of the CAMH agar plate. For MIC measurement, doripenem, imipenem or meropenem Etest strips (bioMérieux) were placed in the middle of the plate. Plates were incubated at 37°C for 24 h before measuring the diameter of the inhibition zone or reading the MIC values.

### Colony wash assay

Plates were prepared by adding 1 ml of LB agar to the wells of a 12‐well plate. Cultures of AB5075 were OD adjusted as previously described using sterile PBS and 5 μl of bacterial suspension was added to the surface of the agar and allowed to dry. Plates were then incubated for 3.5 h at 37°C to allow colony biofilms to form. Once formed, 1 ml of 8.85% ace‐K solution was added to the biofilms. To avoid disruption of the biofilm the solution was gently pipetted down the wall of the well. Controls were either left untreated or were treated with 1 ml of sterile deionised water. Treated biofilms were then incubated statically for a further 1 h at 37°C. Following treatment, the liquid was removed from the wells by pipetting and 1 ml of sterile PBS was added. Each biofilm was resuspended by vigorous pipetting of the PBS. The resuspended biofilm was then diluted and viable cells enumerated.

For colony biofilms treated with gauze, the same protocol was used with the following amendments. Following 3.5 h incubation, 2 cm^2^ pieces of sterile cotton gauze soaked in 8.85% ace‐K solution were added to the surface of the biofilm and gently pressed down to ensure total contact between the gauze and the biofilm. Controls were either left untreated or treated with identical gauze soaked in sterile deionised water. After 1 h of treatment at 37°C, gauze pieces were carefully removed and each biofilm was resuspended and viable cells enumerated as previously described.

### Imaging of pre‐ and post‐treatment biofilms

To image the effect of ace‐k treatment on *A. baumannii*, biofilms were grown on glass microscope slides. Sterile glass slides were placed in sterile tubes. Cultures of AB5075 (OD_600_ 0.5) were added to the tubes so that half of the slide was covered (20 ml total volume) and samples were incubated statically for 18 h at 37°C. Following incubation, slides were removed and biofilms were washed by gently submerging them in sterile deionised water 3 times. Biofilms were then air‐dried in a sterile environment before staining with 200 μl of BacLight reagent (ThermoFisher, UK), prepared as per the manufacturer's guidance. Biofilms were stained for 30 min in a dark, sterile environment. Biofilms were imaged using a Lieca HF14 DM4000 microscope using L5 and CY3 filters. The native LAS AF software was used for image capture. During imaging, five fields of view were visualised and the exact coordinates and imaging parameters of each were noted. The same biofilm was then treated with an 8.85% ace‐K soaked gauze for 1 h before being washed and stained as previously detailed. The same five fields of view were imaged again using the same imaging parameters so that a pre‐ and post‐treatment comparison could be made. Heatmaps and 3‐dimensional representations were prepared as previously described by Pandian *et al* (2021) using the ImageJ “3D Interactive Surface Plot” plugin.

### *Ex vivo* model

Porcine skin was obtained from Fine Food Specialists (London, UK) or GridIron Meat Co. (Skipton, UK). All skin used in *ex vivo* experiments was from the belly of the pig, never frozen, and was free from additives such as salt or additional water. To model an acute laceration, skin was divided into 2.5 cm^2^ sections and placed in individual Petri dishes, and each piece of skin was cut with a scalpel blade. Cuts were 2 cm in length and 2 mm in depth. Skin sections were UV sterilised for 1 h on each side. Following sterilisation, 5 μl of a suspension of AB5075 (OD_600_ 0.05) was added to the wound bed. Skin samples were then incubated for 3.5 h at 37°C to allow wound biofilms to form. Following incubation, 2 cm^2^ pieces of sterile gauze soaked in 1.5 ml of 8.85% ace‐K solution were added to the skin surface. Skin samples were incubated for a further 1 h at 37°C. Following treatment, pieces of gauze were removed and wounds were washed with 1 ml of sterile PBS. Wounds were washed while scraping the wound bed with the pipette tip to dislodge biofilm. This wash was repeated three times with the same volume of PBS to ensure all biofilm was collected. The PBS was then diluted and viable cells were enumerated.

To simulate an acute burn wound skin was cut to a 10 cm^2^ section. An array of 20 steel pins (each with an 8 mm diameter) was heated to 140°C in a heat block for 1 h before being placed on the skin section for 60 s. An even weight was applied to the pin array to ensure consistent burns. Following burning, each burn was excised and added to a 24‐well plate. The burned skin sections were UV sterilised as previously described. Once sterile each burn was inoculated with 5 μl AB5075 suspension (OD_600_ 0.05) and the inoculum was allowed to dry. Inoculated burns were incubated for 3.5 h to allow biofilm to form. A 2 cm^2^ piece of gauze soaked with 1.5 ml of the desired treatment (water, 8.85% ace‐K, unisept, 500 μg/ml polymyxin B sulfate or a combination of polymyxin B and ace‐K) was added to each burn and incubated for a further 1 h. Following treatment, gauze was removed and burn wound biofilms were collected and enumerated as described above.

### Statistical analysis

GraphPad Prism software was used to conduct statistical analysis where appropriate. All experiments were carried out in biological triplicate unless otherwise stated. Full details of all statistical tests can be found in the Materials and Methods sections and corresponding results can be found in the figure legends.

## Author contributions

**Rubén de Dios:** Data curation; formal analysis; investigation; methodology; writing—original draft. **Chris Proctor:** Data curation; formal analysis; investigation; methodology; writing—original draft. **Evgenia Maslova:** Data curation; formal analysis. **Sindija Dzalbe:** Data curation. **Christian J Rudolph:** Data curation; formal analysis; methodology; writing—review and editing. **Ronan R McCarthy:** Conceptualization; data curation; formal analysis; supervision; funding acquisition; investigation; writing—original draft; project administration; writing—review and editing.

## Disclosure and competing interests statement

Brunel University London has two patents covering the therapeutic use of artificial sweeteners and their use to potentiate antibiotic activity.

**The paper explained**

**Problem**

The world is rapidly running out of effective antibiotics to treat bacterial infections making what were once very treatable infections, life‐threatening. There are two main reasons for this, the first is that there has been a major slowdown in the discovery of new antibiotics since the Golden Age of antibiotic discovery in the 1960s and 1970s. The second reason is that mismanagement, and misuse of the antibiotics that are available has led to the emergence of bacteria that are resistant to all known antibiotics. The World Organisation has put some of these pathogens such *Acinetobacter baumannii* and *Pseudomonas aeruginosa* in the spotlight and highlighted that new therapeutics are urgently needed to tackle these pathogens.

**Results**

In this work, we screen a panel of artificial sweeteners frequently used in the food industry assessing their ability to inhibit the growth of a multidrug‐resistant *A. baumannii*, as well as another commonly antibiotic‐resistant pathogen, *P. aeruginosa*. Among them, acesulfame‐K (ace‐K) showed a remarkable impact on both species, which we could later extrapolate to other clinically relevant pathogens. After this, we examined the effect of ace‐K on the global gene expression of *A. baumannii*, observing and validating that it can inhibit the ability of this pathogen to form biofilms, migrate on surfaces and acquire exogenous DNA. Furthermore, carbapenem‐resistant *A. baumannii* exhibited a resensitisation to these last‐line antibiotics in the presence of ace‐K. According to our microscopy experiments, ace‐K produces a growth inhibition by disrupting the bacterial cell envelope, eventually leading to aberrant morphologies, membrane bulging and cell lysis. Finally, we could validate these findings in an *ex vivo* porcine skin model, placing ace‐K as a compound with the potential to be included in novel antimicrobial formulations, particularly for topical use.

**Impact**

The development of novel antimicrobial drugs entails very significant investments in terms of time and money. However, artificial sweeteners, such as ace‐K, have already undergone extensive safety studies and their use is already widely approved by regulatory agencies globally. This work presents ace‐K as a readily available compound that can be used as an antimicrobial or antibiotic adjuvant in novel therapeutic formulations.

## Supporting information



Appendix

Table EV1

Table EV2

Movie EV1

Movie EV2

Movie EV3

Movie EV4

Movie EV5

Dataset EV1

Dataset EV2

Dataset EV3

## Data Availability

The RNA‐seq datasets produced in this study (gene expression dataset series titled "Alteration of global transcription by the artificial sweetener acesulfame‐K in *Acinetobacter baumannii* AB5075") are available at the National Center for Biotechnology Information Gene Expression Omnibus public database under accession number GSE199706 (https://www.ncbi.nlm.nih.gov/geo/query/acc.cgi?acc=GSE199706).
