# Supplementary material for: Artificial sweeteners inhibit multidrug‐resistant pathogen growth and potentiate antibiotic activity
Source: EMBO Mol Med. 2022 Nov 22;15(1):e16397. doi: 10.15252/emmm.202216397 (PMC9832836; doi:10.15252/emmm.202216397)
Supplement: Supplementary file 1 — Appendix [file EMMM-15-e16397-s012.docx]

**Appendix**

**Artificial sweeteners inhibit multidrug resistant pathogen growth and potentiate antibiotic activity**

Rubén de Dios^1^, Chris R Proctor^1^, Evgenia Maslova^1^, Sindija Dzalbe^1^, Christian J. Rudolph^2^ Ronan R. McCarthy^1^

^1^ Division of Biosciences, Department of Life Sciences, Centre of Inflammation Research and Translational Medicine, College of Health, Medicine and Life Sciences, Brunel University London, Uxbridge, UB8 3PH, UK.

^2^ Division of Biosciences, Department of Life Sciences, Centre for Genome Engineering and Maintenance, College of Health, Medicine and Life Sciences, Brunel University London, Uxbridge, UB8 3PH, UK.

**Table of Contents**

**Appendix Figure S1:** Planktonic growth of AS treated *A. baumannii* compared to an equivalent vehicle control after 19 hours.  **p2**

**Appendix** **Figure S2:** Impact of different AS on *P. aeruginosa* planktonic growth after 19 hours.  **p4**

**Appendix** **Figure S3:** Growth of (a) *A. baumannii* AB5075 and (b) *P. aeruginosa* PA14 in M9 minimal media with 2.66% ace-K as the sole carbon source. **p5**

**Appendix Figure S4:** Dispersal of preformed biofilm after treatment with 8.85% ace-K. **p6**

**Appendix Figure S5:** Iron rescue assay in the presence of ace-K 2.66%. **p7**

**Appendix Figure S6:** Biofilm formation assay comparing different mutants in the *csu* genes to the wild type AB5075. **p8**

**Appendix** **Figure S7:** Impacts of ace-K on motility and transformation. **p9**

**Appendix Figure S8:** Membrane permeability.  **p10**

**Appendix** **Figure S9:** Disc diffusion assay comparing different β-lactams in presence or absence of ace-K. **p11**

**Appendix** **Figure S10:** A 3-dimensional representation of fluorescence intensity in SYTO9 stained *A. baumannii* **p12**

**References p13**


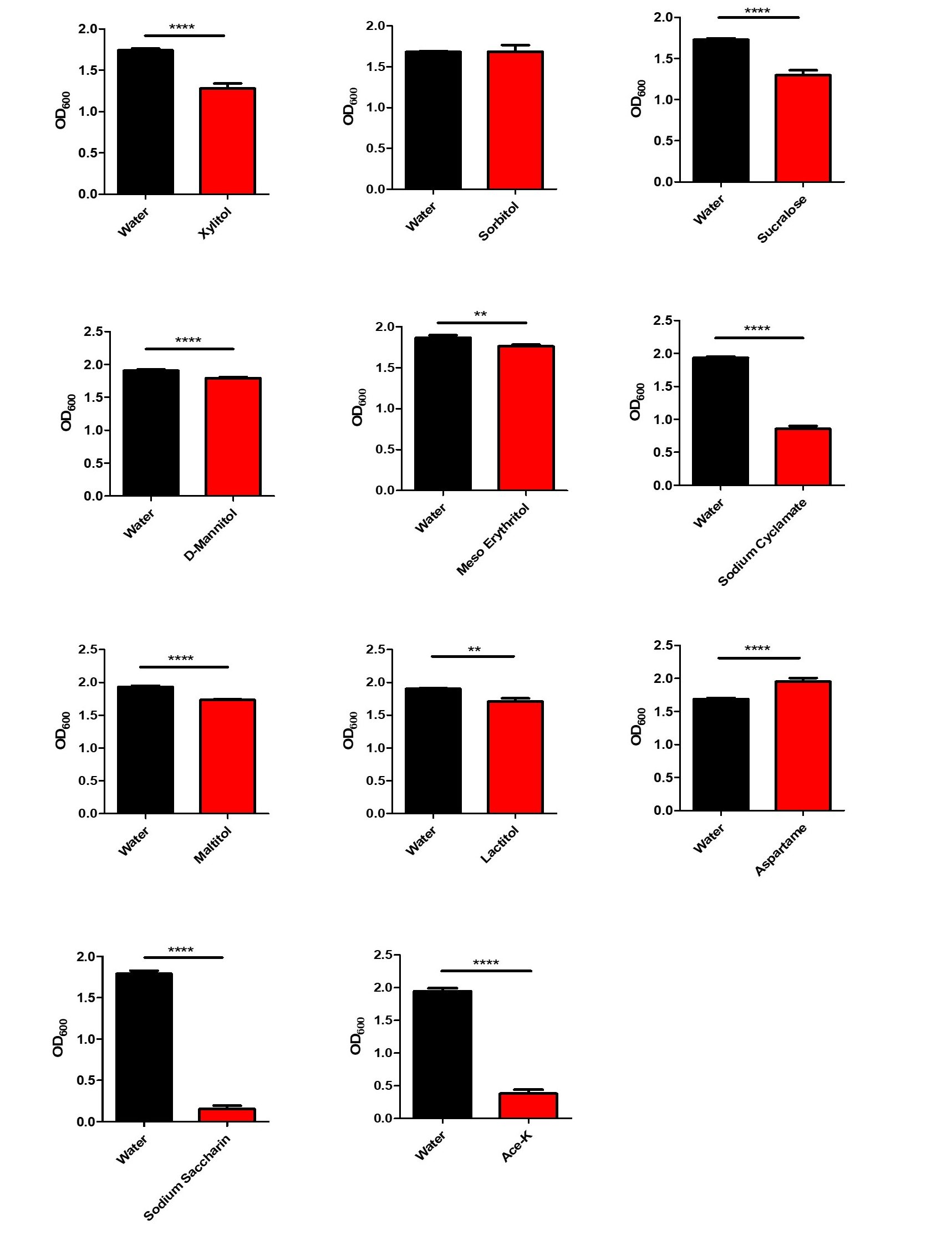


**Appendix Figure S1: Planktonic growth of AS treated *A. baumannii* compared to an equivalent vehicle control after 19 hours.** The majority of tested AS had a negative effect on planktonic growth of *A. baumannii* after 19 hours at a concentration of 2.66%. Sorbitol had no effect on the growth of *A. baumannii*. Data is average of three biological replicates ± S.D. Statistical analysis was by independent t-test between treated samples and their corresponding water control (* p = <0.05, ** p = <0.01, *** p = <0.001, **** p = <0.0001).

**
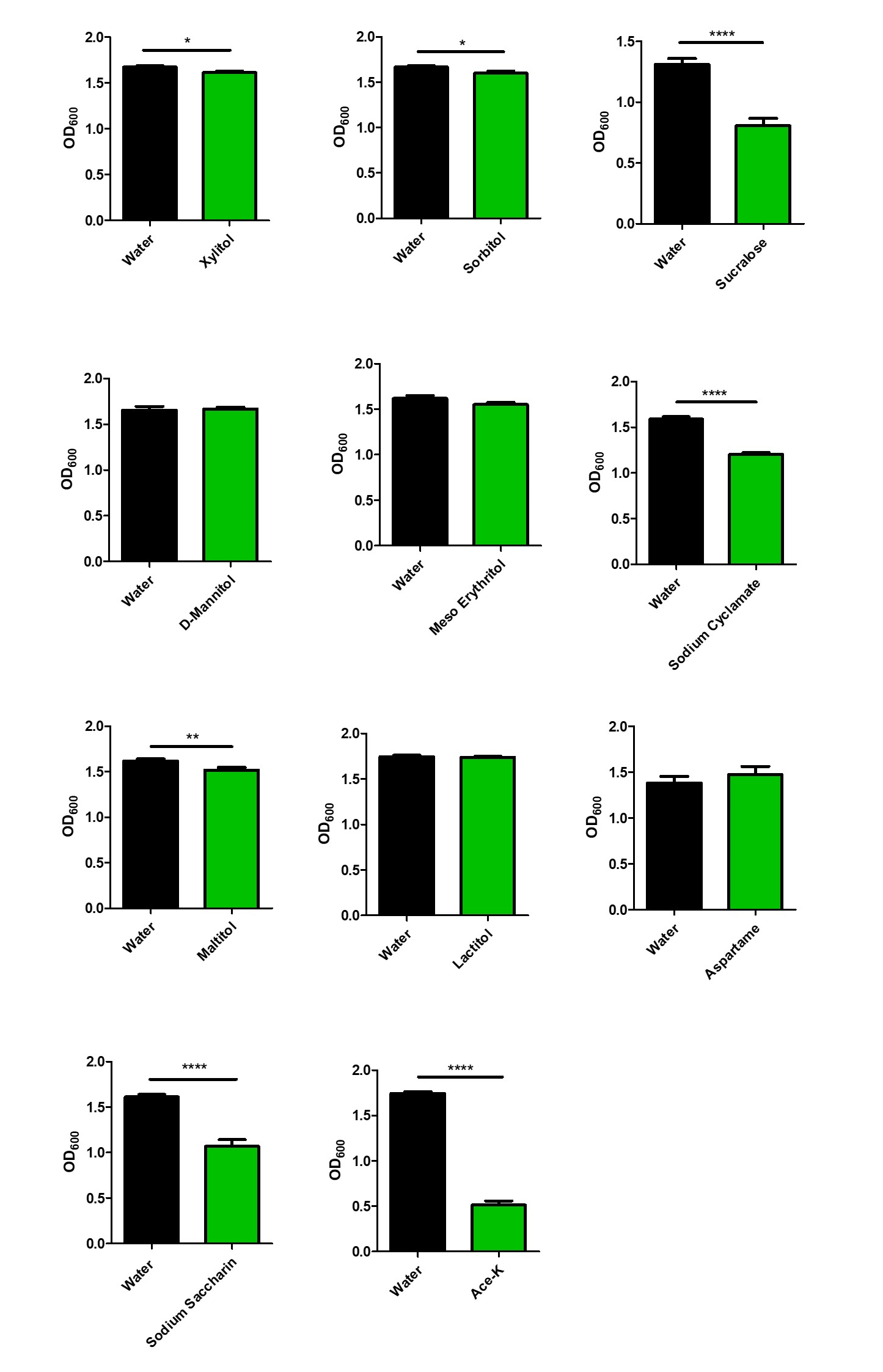
Appendix** **Figure S2: Impact of different AS on *P. aeruginosa* planktonic growth after 19 hours.** The majority of tested AS had a negative effect on planktonic growth of *P. aeruginosa* PA14 after 19 hours at a concentration of 2.66%. D-mannitol, lactitol, erythritol, and aspartame were found to have no significant effects on PA14 growth. Data is average of three biological replicates ± S.D. Statistical analysis was by independent t-test between treated samples and their corresponding water control (* p = <0.05, ** p = <0.01, *** p = <0.001, **** p = <0.0001).

**
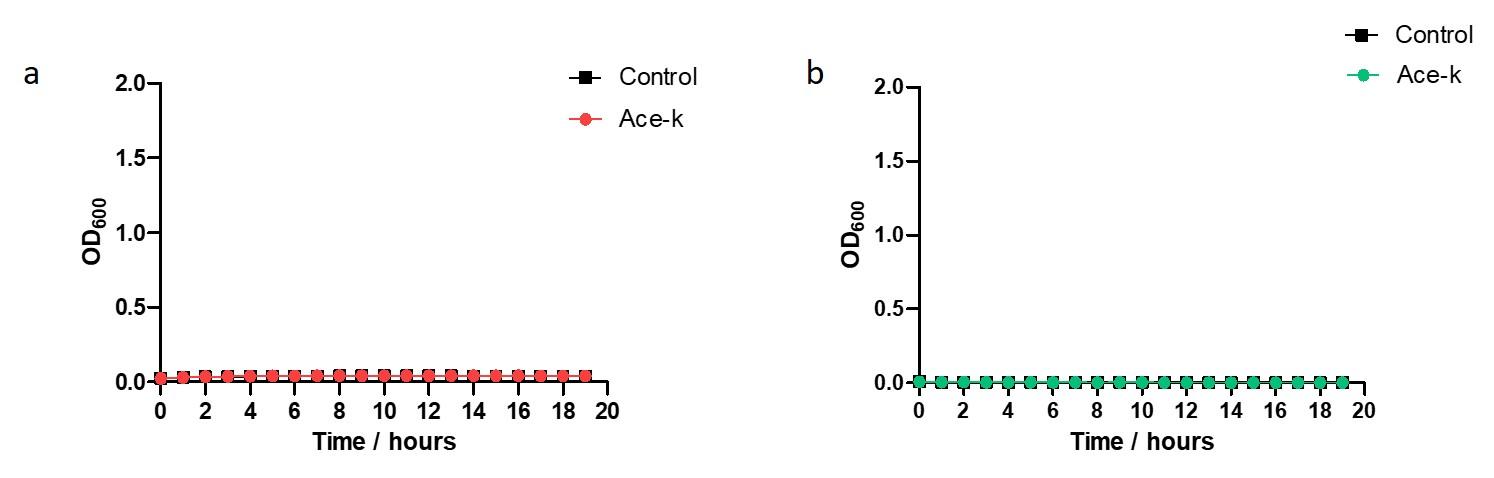
**

**Appendix** **Figure S3: Growth of (a) *A. baumannii* AB5075 and (b) *P. aeruginosa* PA14 in M9 minimal media with 2.66% ace-K as the sole carbon source.** It was shown that neither organism was capable of using the ace-k as a carbon source for planktonic growth. Data is average of 3 biological replicates ± S.D.


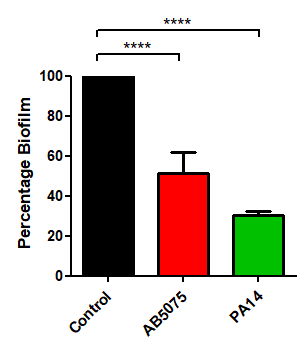


**Appendix Figure S4: Dispersal of preformed biofilm after treatment with 8.85% ace-K.** Treatment of 18 hour AB5075 biofilms with 8.85% ace-k resulted in a 48.8% loss in total biofilm biomass while treatment of 18 h PA14 biofilms resulted in 69.7% reduction in total biomass compared to vehicle controls. Average of three biological replicates ± S.D. are represented. Statistical analysis was by independent t-test (* p = <0.05, ** p = <0.01, *** p = <0.001, **** p = <0.0001).

**
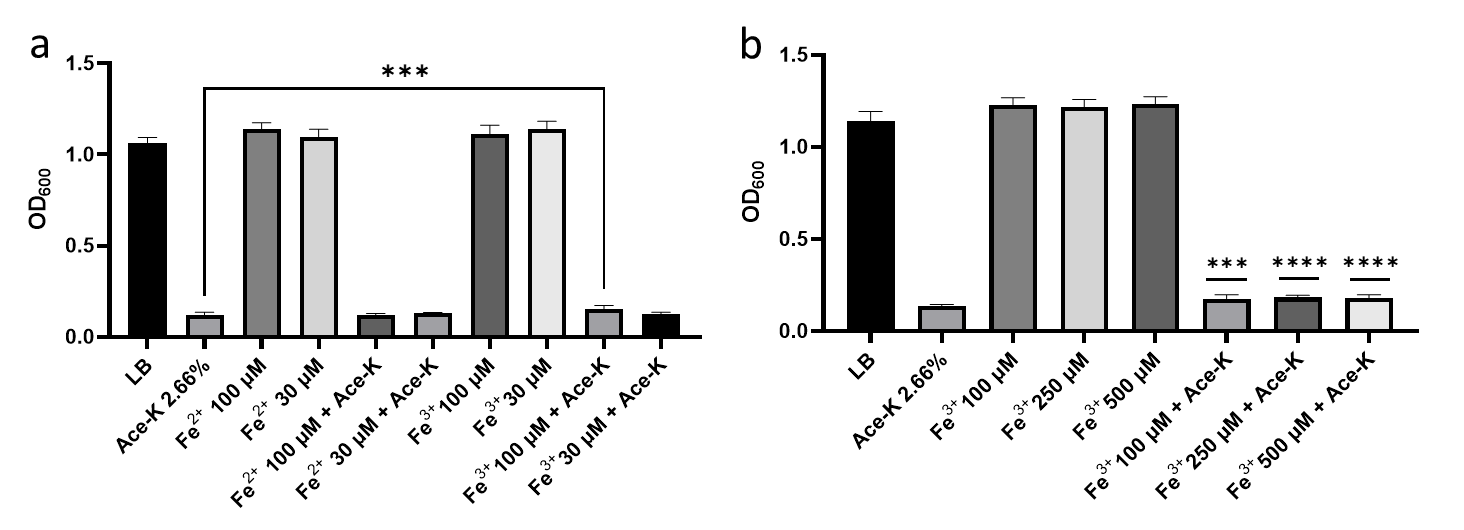
**

**Appendix Figure S5: Iron rescue assay in the presence of ace-K 2.66%.** AB5075 was grown for 24 h in LB broth in presence or absence of ace-K 2.66%. Different concentrations of iron (either Fe^2+^ or Fe^3+^ chloride salts) were added. (a) A first screening was performed using Fe^2+^ and Fe^3+^ at 30 µM and 100 µM concentrations and a significant effect could only be observed when supplementing with Fe^3+^ 100 µM. (b) To assess if there was a dose dependency in the recovery by Fe^3+^ supplementation, we attempted an iron rescue assay with concentrations ranging from 100 µM to 500 µM. However, the results suggest no dose dependency in the recovery from ace-K treatment by addition of Fe^3+^. Average of three biological replicates ± S.D. are represented. Statistical analysis was by independent t-test (*** p = <0.001, **** p = <0.0001).

**
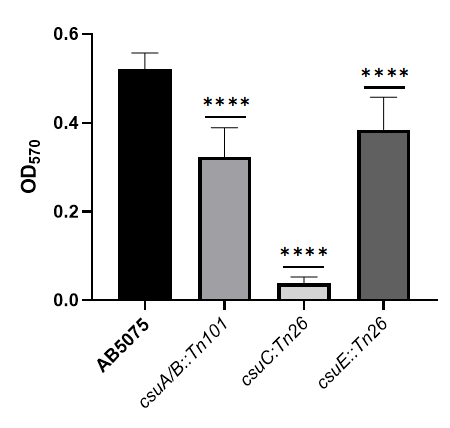
**

**Appendix Figure S6: Biofilm formation assay comparing different mutants in the *csu* genes to the wild type AB5075**. Mutant strains were selected based on the downregulation that the respective genes (*csuA/B, csuC and csuE*) presented in the dRNA-seq analysis. AB5075 and the different mutant strains were grown in LB for 18 h and biofilm formation was quantified using the crystal violet method. The results show a defect in biofilm formation in all the mutants compared to the wild type, indicating their downregulation in the presence of ace-K leads to a decreased biofilm formation. Average of three biological replicates ± S.D. are represented. Statistical analysis was by independent t-test (**** p = <0.0001).

**
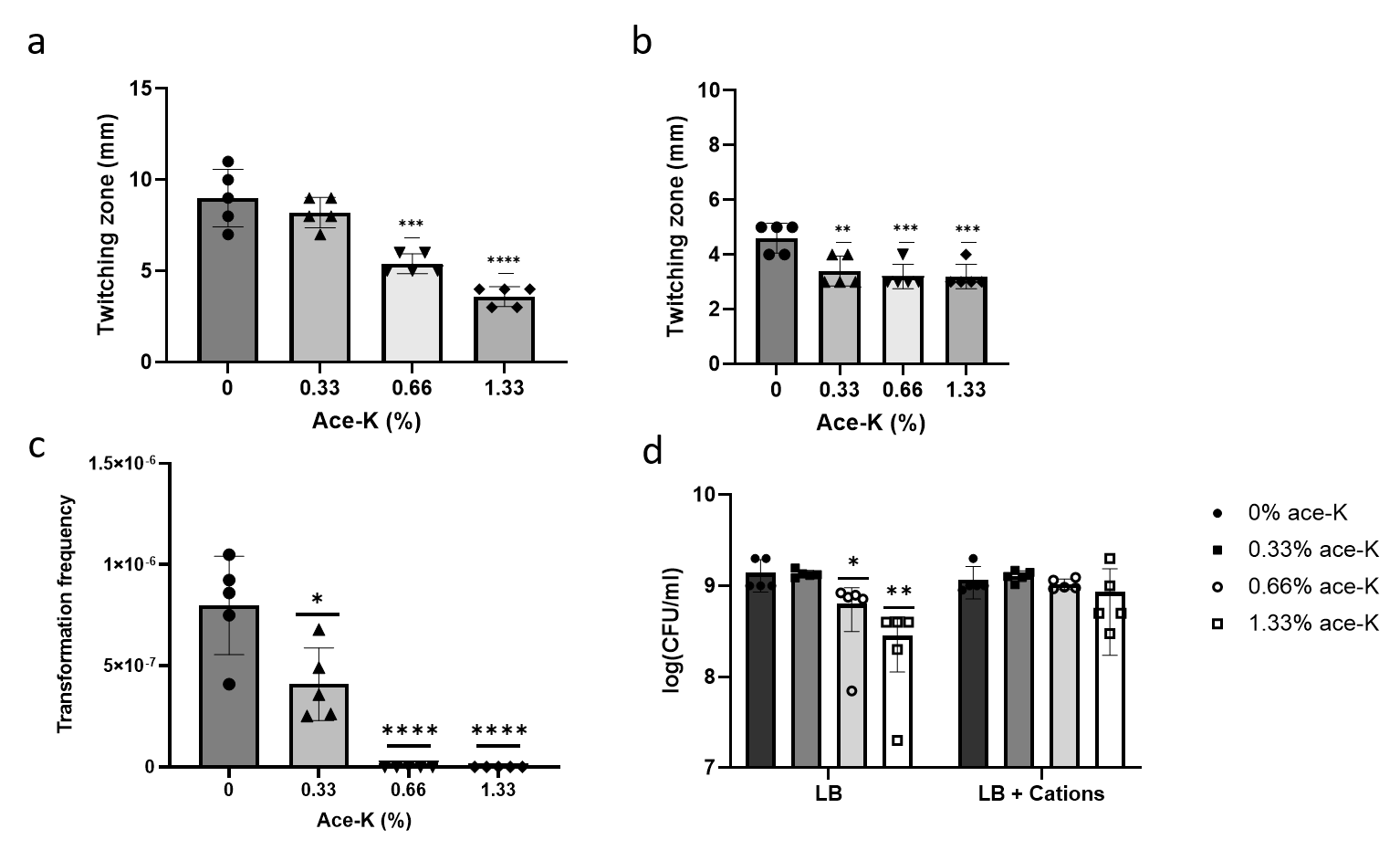
**

**Appendix** **Figure S7: Impacts of ace-K on motility and transformation.** (a and b) show the results of twitching motility assays in a range of concentrations of ace-K for *A. baumannii* AB0057 and BAA 747, respectively. (c) Natural transformation results in LB supplemented with cations (CaCl_2_ 2 mM and MgSO_4_ 1 mM) in the presence of a range of concentrations of ace-K. (d) Viable cell counts measured in the natural transformation assay (with and without cations) in the presence of a range of ace-K concentrations. The viability was not affected in the presence of cations with any ace-K concentration, although transformability was completely abolished above 0.66% ace-K. This indicates the decrease in transformability is not due to a decrease in viability. Results are represented as averages of five biological replicates ± S.D. Statistical analysis was by independent t-test between treated samples and their corresponding water control (* p = <0.05, ** p = <0.01, *** p = <0.001, **** p = <0.0001).

**
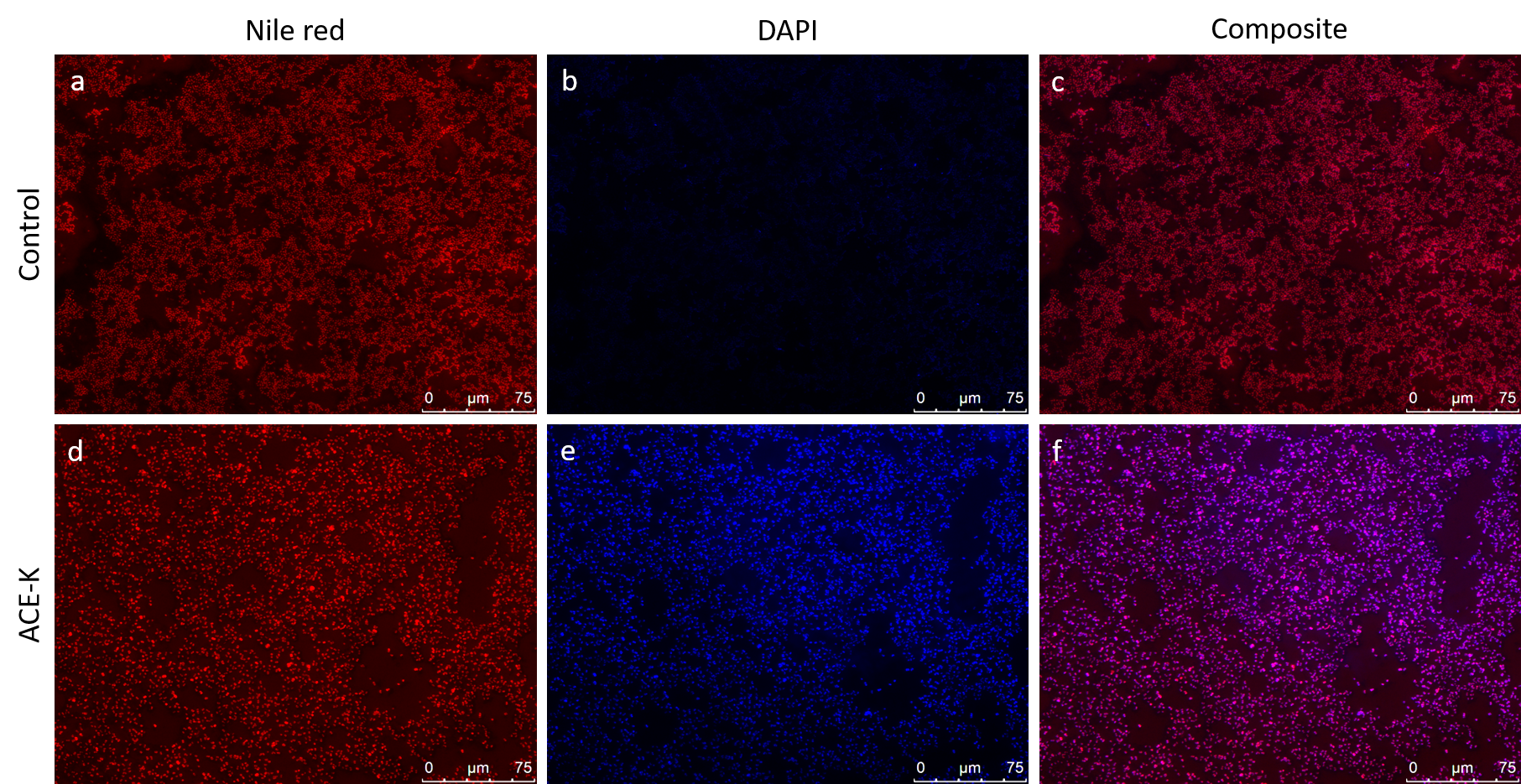
**

**Appendix Figure S8: Membrane permeability.** Differential fluorescence staining of AB5075 with Nile Red and DAPI. When grown in LB with a vehicle control the cell membrane stains as expected with Nile Red and no significant uptake of DAPI is seen. Cell morphology is as expected in control samples. When grown in the presence of 1.33% ace-K the cell membrane is still stained with Nile Red. However, there is significant staining of chromosomal DNA with DAPI indicating a more permeable membrane. Images shown are representative of two biological replicates each performed in technical triplicate.


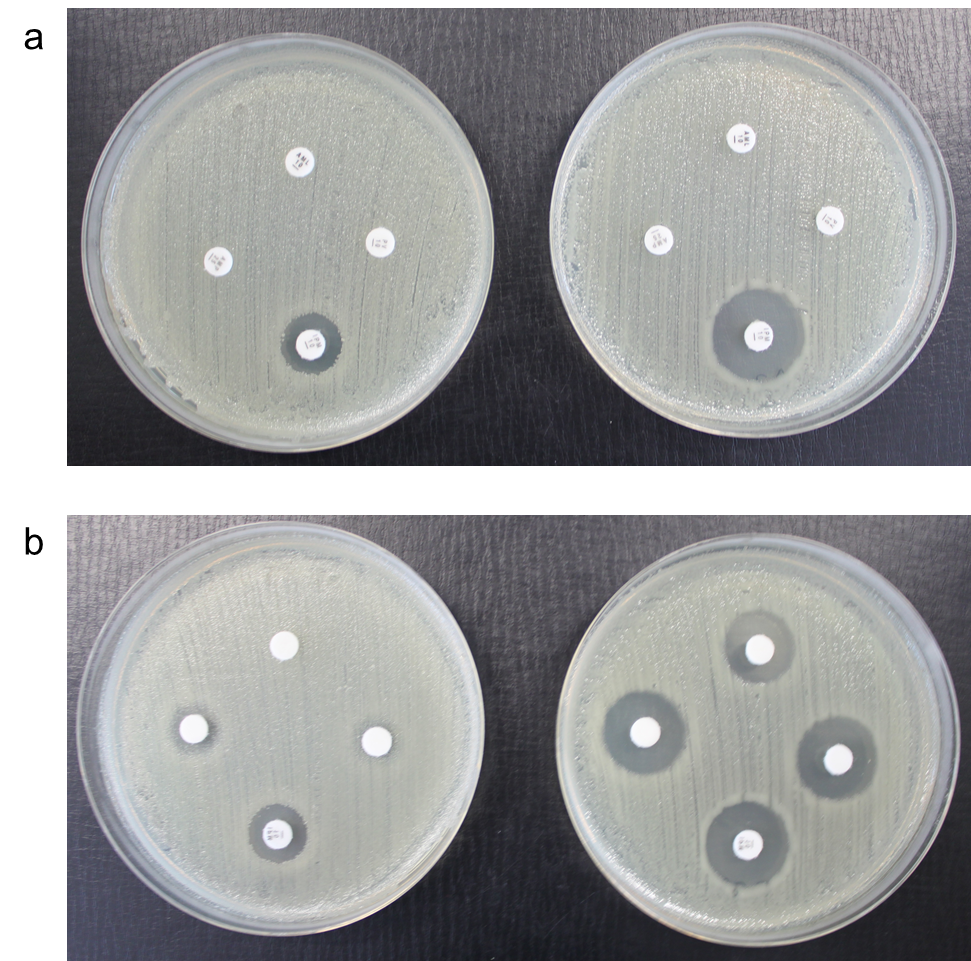


**Appendix** **Figure S9: Disc diffusion assay comparing different β-lactams in presence or absence of ace-K.** The results indicate that the resensitising effect of ace-K is more specific to carbapenems as compared to other β-lactam antibiotics, since much higher concentrations of ampicillin, amoxicillin and penicillin V were needed for achieving a similar resentisation to that observed with imipenem. The assay was performed in cation-adjusted Mueller-Hinton agar supplemented with 2.2% ace-K (right) or a water control (left). The antibiotic discs used in this assay were, clockwise from the bottom: A) imipenem (10 µg), ampicillin (25 µg) amoxicillin (10 µg), penicillin V (10 µg); B) imipenem (10 µg), ampicillin (1000 µg) amoxicillin (1000 µg), penicillin V (1000 µg). The figure shows a representative picture out of three biological replicates.

**
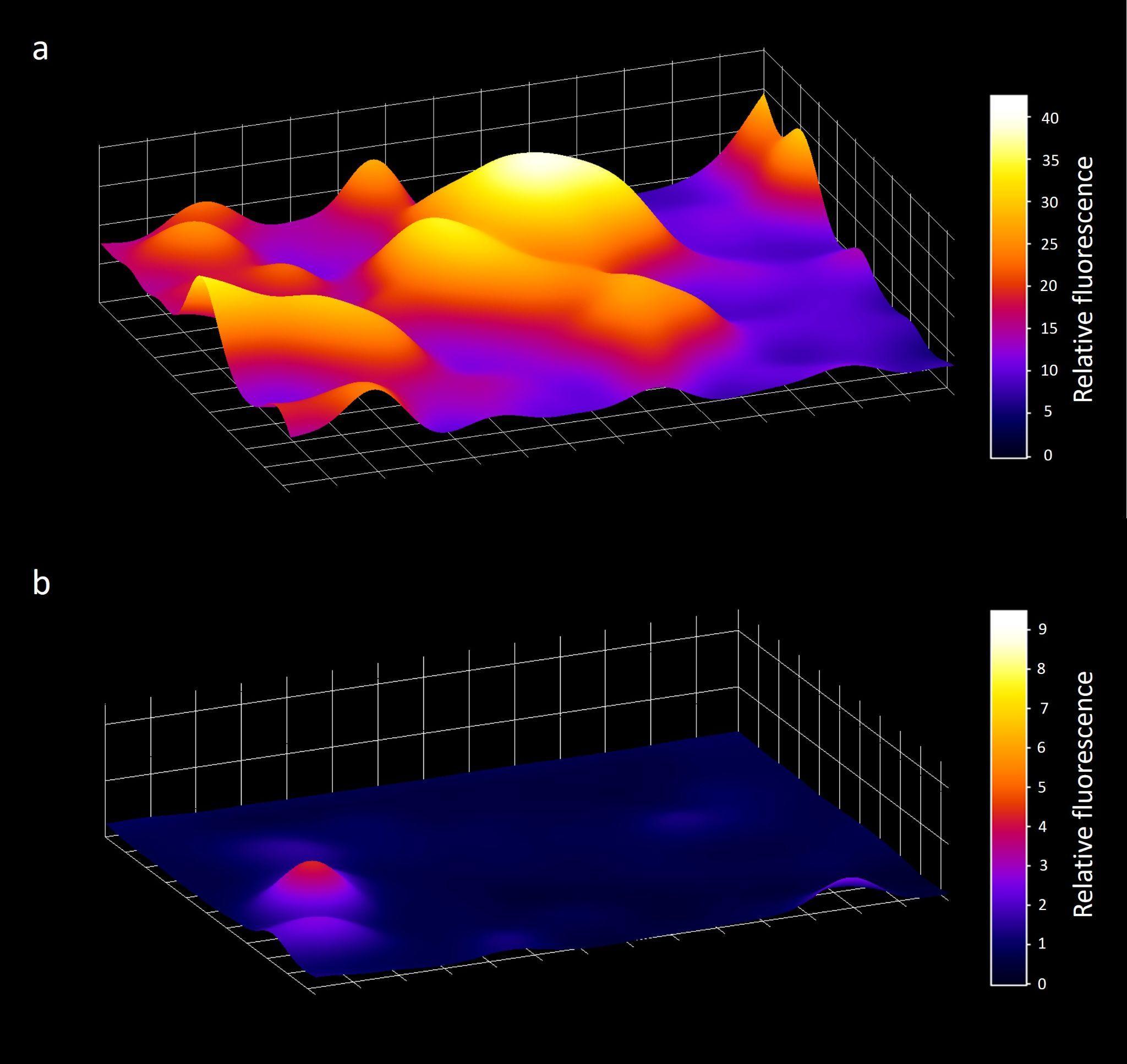
**

**Appendix** **Figure S10: A 3-dimensional representation of fluorescence intensity in SYTO9 stained *A. baumannii* (**a) before treatment and (b) following 1 hour treatment with 8.85% ace-K soaked gauze. It was shown that when the same field of view was imaged before and after treatment with ace-K that relative fluorescence significantly decreased. This is indicative of a reduction in the number of viable cells. Images are representative of 5 fields of view.

**References**

Bachmann (1996) Derivations and Genotypes of Some Mutant Derivatives of *Escherichia coli* K-12. In *Escherichia coli and Salmonella*, 2nd Edition Neidhardt (ed), pp 2460-2488. Washington DC: ASM Press.

Dortet L, Bréchard L, Poirel L, Nordmann P (2014) Rapid detection of carbapenemase-producing Enterobacteriaceae from blood cultures. *Clin Microbiol Infect* 20: 340-344.

Dortet L, Poirel L, Abbas S, Oueslati S, Nordmann P (2015) Genetic and biochemical characterization of FRI-1, a carbapenem-hydrolyzing class A β-lactamase from *Enterobacter cloacae.* *Antimicrob Agents Chemother* 59: 7420-7425.

Furniss RCD, Kadeřábková N, Barker D, Bernal P, Maslova E, Antwi AAA, McNeil HE, Pugh HL, Dortet L, Blair JMA *et al* (2022) Breaking antimicrobial resistance by disrupting extracytoplasmic protein folding. *eLife,* 11: e57974.

Hanahan D (1983) Studies on transformation of Escherichia coli with plasmids. *J Mol Biol*. 166: 557-80.

Martínez-García E, de Lorenzo V (2011) Engineering multiple genomic deletions in Gram-negative bacteria: analysis of the multi-resistant antibiotic profile of *Pseudomonas putida* KT2440. *Environ Microbiol*. 13: 2702-16.

Nordmann P, Poirel L, Dortet L (2012) Rapid detection of carbapenemase-producing *Enterobacteriaceae* *Emerg Infect Dis* 18: 1503-1507.

Schroth MN, Cho JJ, Green SK, Kominos SD (1977) Epidemiology of *Pseudomonas aeruginosa* in agricultural areas. In *Pseudomonas Aeruginosa: Ecological Aspects and Patient Colonization*, Young VM (ed) pp1-29 New York: Raven Press.

Wang X, Possoz C, Sherratt DJ (2005) Dancing around the divisome: asymmetric chromosome segregation in *Escherichia coli*. *Genes Dev*. 19: 2367-77.
