# Supplementary material for: Artificial sweeteners inhibit multidrug‐resistant pathogen growth and potentiate antibiotic activity
Source: EMBO Mol Med. 2022 Nov 22;15(1):e16397. doi: 10.15252/emmm.202216397 (PMC9832836; doi:10.15252/emmm.202216397)
Supplement: Supplementary file 5 — Movie EV2 [file EMMM-15-e16397-s008.zip › Movie EV2 Legend.docx]

**Movie EV2**: Time lapse of *A. baumannii* AB5075 cells growing in the presence of 2.66% ace-K. 1.5x magnification used compared to Movie EV1 to enable greater visualisation of membrane bulges and cell morphology changes. For full details on experimental set up see Methods.
