# Supplementary material for: Artificial sweeteners inhibit multidrug‐resistant pathogen growth and potentiate antibiotic activity
Source: EMBO Mol Med. 2022 Nov 22;15(1):e16397. doi: 10.15252/emmm.202216397 (PMC9832836; doi:10.15252/emmm.202216397)
Supplement: Supplementary file 8 — Movie EV5 [file EMMM-15-e16397-s010.zip › Movie EV5 Legend.docx]

**Movie EV5**: Side by side time lapse of *E. coli* MG1655 (JD1709 pCP8) cells growing in the absence and the presence of ace-K. For full details on experimental set up see Methods.
