# Supplementary material for: Artificial sweeteners inhibit multidrug‐resistant pathogen growth and potentiate antibiotic activity
Source: EMBO Mol Med. 2022 Nov 22;15(1):e16397. doi: 10.15252/emmm.202216397 (PMC9832836; doi:10.15252/emmm.202216397)
Supplement: Supplementary file 12 [file EMMM-15-e16397-s011.pdf]

EMBO Molecular Medicine

Artificial sweeteners inhibit multidrug resistant pathogen growth and potentiate antibiotic activity

Ruben de Dios, Chris Proctor, Evgenia Maslova, Sindija Dzalbe, Christian J. Rudolph, and Ronan McCarthy
DOI: 10.15252/emmm.202216397

Corresponding author: Ronan McCarthy (ronan.mccarthy@brunel.ac.uk)

Review Timeline:

Submission Date:	2nd Jun 22
Editorial Decision:	12th Jul 22
Revision Received:	18th Oct 22
Editorial Decision:	27th Oct 22
Revision Received:	28th Oct 22
Accepted:	28th Oct 22

Editor: Zeljko Durdevic

Transaction Report:

(Note: With the exception of the correction of typographical or spelling errors that could be a source of ambiguity, letters and reports are not edited. Depending on transfer agreements, referee reports obtained elsewhere may or may not be included in this compilation. Referee reports are anonymous unless the Referee chooses to sign their reports.)

12th Jul 2022

Dear Dr. McCarthy,

Thank you for the submission of your manuscript to EMBO Molecular Medicine, and please accept my apologies for the delay in getting back to you. We have received feedback from two of the three reviewers who agreed to evaluate your manuscript. Given that referee #2 will unfortunately not be able to return his/her report in a timely manner, and that both referees #1 and #3 gave similar recommendation, we prefer to make a decision now in order to avoid further delay in the process. Should referee #2 provide a report, we will send it to you, with the understanding that we will not ask for an additional revision. As you will see from the reports below, the referees acknowledge the interest and novelty of the study but also raise serious and partially overlapping concerns that should be addressed in a major revision. Focus of the revision should be on providing a positive control in the wound model, experimental validation of the RNAseq data to show its significance and providing some insight of how Ace-K disrupts the membrane.

Addressing the reviewers' concerns in full will be necessary for further considering the manuscript in our journal, and acceptance of the manuscript will entail a second round of review. EMBO Molecular Medicine encourages a single round of revision only and therefore, acceptance or rejection of the manuscript will depend on the completeness of your responses included in the next, final version of the manuscript. For this reason, and to save you from any frustrations in the end, I would strongly advise against returning an incomplete revision.

We would welcome the submission of a revised version within three months for further consideration. Please let us know if you require longer to complete the revision.

Please use this link to login to the manuscript system and submit your revision: https://embomolmed.msubmit.net/cgi-bin/main.plex

I look forward to receiving your revised manuscript.

Yours sincerely,

Zeljko Durdevic

Zeljko Durdevic
Editor
EMBO Molecular Medicine

***** Reviewer's comments *****

Referee #1 (Comments on Novelty/Model System for Author):

Some of this work replicates or builds modestly on what has been done before (e.g. Yu et al., 2022 ISME J) has shown the impact of sweeteners on bacterial membrane integrity. However, other aspects are novel (antibiotic potentiation) or contradict existing literature (some of the gene regulation data). I'm not sure of the value of the wound model data since there is no comparison to a currently used topical agent.

Referee #1 (Remarks for Author):

The manuscript from de Dios et al describes the effect of artificial sweeteners on bacterial growth and various phenotypic properties related to virulence and antibiotic resistance, with a focus on Ace-K.

The manuscript is well written and logical and refers to appropriate previous work where relevant. The most interesting findings are that artificial sweetener Ace-K reduces bacterial growth, horizontal gene transfer and potentiates the activity of some clinically relevant antibiotics. This is likely to be of broad interest to the field.

My main issue is around the novelty of the findings since Ace-K has already been shown to increase membrane permeability and alter the expression of relevant genes in Actinobacter (e.g. Yu et al., 2022 ISME J). The finding that Ace-K blocks transformation is in contrast to this work, as are the findings related to down-regulation of pili. However, it's not clear why this is e.g. if these effects occur at a single concentration of Ace-K as there's no dose-response data or if this effect is strain-specific (previous work seems to use lower concentrations). However, the data showing potentiation of antibiotics and potential applications as a wound dressing are novel and potentially impactful.

Additional major points:

Line 153: It is a big stretch to call PA14 a clinical isolate as it's been used in laboratory studies for many years.

Lines 153-157: Please comment in the text how the concentrations of sweeteners were selected. Was it based on those in food/ the gut?

Figure 1. It's not clear to me why the authors show a full array of data for A. baumannii but nothing for P. aeruginosa, which is in the supplementary file. I would prefer to see the key results for both pathogens in the main figures (i.e. Ace-K for both pathogens). Data for sweeteners that have no effect on growth can be placed in the supplementary file. It's also not clear what the difference is between the graphs in supplementary figures S1 and S3 and those in figure 1 and S2. It looks like S1 and S3 represent a single time point that is subjected to statistical analyses. However, it would be better to do statistical analyses on the time course data (e.g. 2-way repeated measures ANOVA).
Figure 2. It is inappropriate to use a series of t-tests here (unless corrected for multiple comparisons). Whilst the comparisons are between 2 data points, the assay consists of multiple data points. Therefore, a test such as a 2-way ANOVA should be used.

The biofilm inhibition assay is interesting in that some concentrations that only partially inhibit growth (e.g. 1.77% for P. aeruginosa) have a much greater inhibitory effect on biofilm formation i.e. that biofilm inhibition isn't simply a function of growth inhibition (which is likely the case at higher concentrations of Ace-K). However, what would really be exciting is if Ace-K could eradicate pre-formed biofilms since this is a major clinical challenge. There is some attempt to do this in the ex vivo model but it's not clear if biofilm is eradicated or just that some of the bacteria are killed.

I understand why the authors pursued T4P for follow up analysis after the transcriptional study but having previously shown an impact of Ace-K on biofilm, it seems odd that there was no attempt to link the transcriptional data to biofilm inhibition in functional assays. It's entirely feasible that T4P contribute to biofilm formation via their role in adhesion (e.g. Colquhoun and Rather 2020 Front Cell Infect Micro). Indeed, the authors comment on the potential effect of down-regulation of pili and gene encoding Bap but don't attempt to show a causative link to inhibition of biofilm.

Figure 3. Why are the concentrations used here different from the growth inhibition assays? This makes it hard to understand the potential confounding effect of growth inhibition. For figure 3c I think it important to show growth of bacteria in the absence of antibiotic selection i.e. at the concentrations of Ace-K used, there are similar numbers of viable bacteria that could grow (I suggest to repeat the experiment without antibiotic selection and show equal numbers of bacteria in all conditions used). Also, the term 'cations' isn't specific. Which cations and how much of them should be made clear in the main text.

Lines 276-284 are interesting but it's not clear what it adds to the work since there is no attempt to determine the functional significance of the findings. Does iron supplementation relieve growth inhibition caused by Ace-K? Overall, whilst I understand why the authors feel they should include a large description of the transcriptomics work, its value is really only in leading to the effect of Ace-K on transformation and membrane disruption, with the rest of this section being speculative in the absence of any functional follow up work.

Lines 309-311. It would be useful to give some examples of the genes that are being referred to and why this is indicative of membrane stress. How do these gene profiles fit with other membrane stressors, particularly EDTA which also causes membrane permeabilization via cation destabilisation.

The wound model data are not hugely convincing and would benefit from a comparator treatment. How does Ace-K compare to alternative wound cleaning antiseptics? Alternatively, does Ace-K potentiate antibiotics in the context of the wound model?
It appears that Ace-K is destabilising the membrane by disrupting the cation bridges between LPS molecules, most likely by chelating cations (since growth was restored by cation supplementation). How will this impact wound healing since cations are required for the integrity of cell-cell interactions and attachment to the connective tissue?

Minor points:

Lines 66-70 is certainly true but would benefit from supporting references.
Lines 94-97 are a little confusing. If phytochemicals are promising, why look elsewhere? Why not develop the phytochemicals?
Line 103 - what is meant by 'intensive'?
Line 141: remove 'the'.
Figure 2. The text describing figure 2 falls into two sections with separate headings. Therefore, I would either merge the text or split the figure to make it easier to follow for the reader.
Lines 325-328. I don't understand why the authors find it remarkable that Ace-K inhibits the growth of multiple species of bacteria, since they have already shown an effect on two different species and effects have been reported already in other bacteria.

Referee #3 (Remarks for Author):

This study shows that artificial sweeteners could show an inhibitory effect on the growth and virulence of infectious pathogens. This is a novel and interesting finding. Here are some concerns.

1. Most of all studies are focused on the Ace-K but the saccharin also has a strong inhibitory effect on the growth of bacteria. Does saccharin have a similar effect as Ace-K on mobility, biofilm formation, and membrane stability? Some key experiments should be done to address for making clear that these effects are specific for Ace-K or for all artificial sweeteners?

2. It is very interesting to see a positive result in the ex vivo model, however, since there is lacking a positive control (an antibiotic). So, it is too difficult to compare the results to see whether it is feasible to be applied in clinical in the future. Also, the dose of 8.85% seems to be a very high dose and was not tested in previous studies (in inhibitory effect, mobility, biofilm).

3. Why Ace-K could destabilize the membrane, what are possible explanations? Will it affect mammalian cell membrane stability?

4. What are IC50s for each sweetener on inhibitory effect? these should be labeled on the Figures.

5. Some functional experiments are needed for dRNA-seq validation. The authors found the pathways were responsible for the Ace-K effect. However, genetic evidence was lacking. Whether pil or com overexpression strains could resist the Ace-K effect?

6. Whether it is possible for testing the Ace-K role of sensitizing MRS to antibiotics in an animal model?

***** Reviewer's comments ***** Responses in Black

Referee #1 (Comments on Novelty/Model System for Author):

Some of this work replicates or builds modestly on what has been done before (e.g. Yu et al., 2022 ISME J) has shown the impact of sweeteners on bacterial membrane integrity. However, other aspects are novel (antibiotic potentiation) or contradict existing literature (some of the gene regulation data). I'm not sure of the value of the wound model data since there is no comparison to a currently used topical agent.

Referee #1 (Remarks for Author):

The manuscript from de Dios et al describes the effect of artificial sweeteners on bacterial growth and various phenotypic properties related to virulence and antibiotic resistance, with a focus on Ace-K. The manuscript is well written and logical and refers to appropriate previous work where relevant. The most interesting findings are that artificial sweetener Ace-K reduces bacterial growth, horizontal gene transfer and potentiates the activity of some clinically relevant antibiotics. This is likely to be of broad interest to the field.

My main issue is around the novelty of the findings since Ace-K has already been shown to increase membrane permeability and alter the expression of relevant genes in Actinobacter (e.g. Yu et al., 2022 ISME J). The finding that Ace-K blocks transformation is in contrast to this work, as are the findings related to down-regulation of pili. However, it's not clear why this is e.g. if these effects occur at a single concentration of Ace-K as there's no dose-response data or if this effect is strain-specific (previous work seems to use lower concentrations). However, the data showing potentiation of antibiotics and potential applications as a wound dressing are novel and potentially impactful.

We thank the reviewer for their insightful and supportive comments and for commenting on the impactfulness of the work. We have taken each of the comments on board and addressed each extensively including through performing additional experiments. We are in no doubt that these changes have significantly strengthened the manuscript and as a result the potential for impact.

We have performed several additional experiments to address this comment specifically. We have demonstrated that the down regulation of pili is dose dependent firstly by reperforming the twitching motility assay and the transformation assay at a range of concentrations and observing a clear dose dependent response (Fig.4cd). We have also demonstrated that this effect is not strain specific by performing twitching assays using two additional strains of *A. baumannii,* BAA-747 and AB00057 (Supplementary Figure 7ab). To further confirm the down regulation of pili, we generated a *pilA* promoter ‘*gfp* transcriptional fusion. This reporter further validated the RNASeq data by confirming the dose dependent effect of ace-K on *pilA* expression (Fig. 4b). This data highlights the therapeutic potential of ace-K as a virulence limiting agent and demonstrates that the effects of ace-K are dose dependent and non-strain specific. This additional data is now discussed in lines 285-300.

Additional major points:

Line 153: It is a big stretch to call PA14 a clinical isolate as it's been used in laboratory studies for many years.
While the original isolate of PA14 was from an infected wound, which has led to this strain being preferentially used in studies profiling wound infection, we do acknowledge that it has likely undergone significant adaptation over years of routine lab culture, the text has been altered to indicate this. Line 154.

Lines 153-157: Please comment in the text how the concentrations of sweeteners were selected. Was it based on those in food/the gut?

During our initial screening phase of this study we normalised the concentrations based on solubility such that each sweetener was at the same concentration (with the exception of aspartame) so that their effects could be comparable. This has now been clarified in the main text specifically in lines 157-160.

Figure 1. It's not clear to me why the authors show a full array of data for A. baumannii but nothing for P. aeruginosa, which is in the supplementary file. I would prefer to see the key results for both pathogens in the main figures (i.e. Ace-K for both pathogens). Data for sweeteners that have no effect on growth can be placed in the supplementary file. It's also not clear what the difference is between the graphs in supplementary figures S1 and S3 and those in figure 1 and S2. It looks like S1 and S3 represent a single time point that is subjected to statistical analyses. However, it would be better to do statistical analyses on the time course data (e.g. 2-way repeated measures ANOVA).

Figure 2. It is inappropriate to use a series of t-tests here (unless corrected for multiple comparisons). Whilst the comparisons are between 2 data points, the assay consists of multiple data points. Therefore, a test such as a 2-way ANOVA should be used.

We thank the reviewer for this insightful comment and fully agree that it makes more sense to move the *P. aeruginosa* growth curve data to the main text. Reanalysis of the growth curve data has been undertaken as suggested by the reviewer using 2-way repeated measures ANOVA for both AB5075 and PA14. Data on the effect of artificial sweeteners on the growth kinetics on PA14 have now also been included in the main text (Line 163-166). We sought to include the growth curves to show potential lags in growth that occurred with some sweeteners while the endpoint analysis was to show that a significant effect was seen at this specific time point (19 hours). We have now clarified this in the text also (Line 166-168). For the MBIC assays, the statistics have also been redone using a two-way repeated ANOVA.

The biofilm inhibition assay is interesting in that some concentrations that only partially inhibit growth (e.g. 1.77% for P. aeruginosa) have a much greater inhibitory effect on biofilm formation i.e. that biofilm inhibition isn't simply a function of growth inhibition (which is likely the case at higher concentrations of Ace-K). However, what would really be exciting is if Ace-K could eradicate pre-formed biofilms since this is a major clinical challenge. There is some attempt to do this in the ex vivo model but it's not clear if biofilm is eradicated or just that some of the bacteria are killed.

We thank the reviewer for raising this excellent point and fully agree that the eradication of preformed biofilms would be exciting and address a major clinical challenge. Based on the reviewer's suggestion we assessed the ability of an ace-K solution to eradicate preestablished biofilms and demonstrated that an 8.85% ace-k solution does indeed reduce total biomass in preformed biofilms of both AB5075 and PA14 (Supplementary Figure S4). This is a particularly important finding from a clinical perspective and this is now discussed in lines 211-215.

I understand why the authors pursued T4P for follow up analysis after the transcriptional study but having previously shown an impact of Ace-K on biofilm, it seems odd that there was no attempt to link the transcriptional data to biofilm inhibition in functional assays. It's entirely feasible that T4P contribute to biofilm formation via their role in adhesion (e.g. Colquhoun and Rather 2020 Front Cell Infect Micro). Indeed, the authors comment on the potential effect of down-regulation of pili and gene encoding Bap but don't attempt to show a causative link to inhibition of biofilm.

The reviewer raises a very valid point which we have now addressed by clarifying the link between the differential expression of genes associated with biofilm formation in the presence of ace-K and their subsequent role in the biofilm inhibition phenotype in the main text. For example, we have already validated the reduced expression of *pilA* in the presence of ace-k using our transcriptional fusion (Figure 4b), however the role of PilA in biofilm formation is substrate specific and it has previously

been shown that biofilm formation on stainless steel is impacted in a *pilA* mutant but attachment to other substrates such as glass is not (Ronish et al., 2019, PMID: 30413536:). We tested the influence of *pilA* transposon mutant on biofilm formation under the conditions used in this study and confirmed that it does not have a diminished biofilm phenotype. This suggests that the down regulations of the T4P is having minimal effect on biofilm but is responsible for the impact of ace-K on twitching motility. We next assessed the *csu* pili, which have been shown to influence biofilm formation (Moon *et al.,* 2017, PMID: 28674047, Luo *et al.,* 2015, PMID: 25888221, Pakharukova *et al.,* 2018, PMID: 29735695). We confirmed the link between the *csu* pili and biofilm formation under our conditions by confirming that transposon mutants in *csuA/B, csuC* and *csuE* (all down regulated significantly in our RNA-Seq data set) all have diminished biofilm formation (Supplementary Figure S6) supporting the hypothesis that ace-K is disrupting biofilm through the downregulation of the *csu* pili. This data is discussed in lines 248-263.

Figure 3. Why are the concentrations used here different from the growth inhibition assays? This makes it hard to understand the potential confounding effect of growth inhibition. For figure 3c I think it important to show growth of bacteria in the absence of antibiotic selection i.e. at the concentrations of Ace-K used, there are similar numbers of viable bacteria that could grow (I suggest to repeat the experiment without antibiotic selection and show equal numbers of bacteria in all conditions used). Also, the term 'cations' isn't specific. Which cations and how much of them should be made clear in the main text.

We apologise for the lack of clarity here and we have now addressed this in the main text. Specifically, 1.33% was used to minimise the transcriptional noise that could occur due to major growth inhibitory effects seen at higher ace-K concentrations (Line 220-222). Viability data is available and is now shown in Supplementary Figure 7d demonstrating no impact on cell viability in the cation conditions. Cation details have now also been specified in line 314-315.

Lines 276-284 are interesting but it's not clear what it adds to the work since there is no attempt to determine the functional significance of the findings. Does iron supplementation relieve growth inhibition caused by Ace-K? Overall, whilst I understand why the authors feel they should include a large description of the transcriptomics work, its value is really only in leading to the effect of Ace-K on transformation and membrane disruption, with the rest of this section being speculative in the absence of any functional follow up work.

We appreciate the reviewers comment here and in particular their insight on the potential role of iron. Based on this suggestion we have conducted iron rescue assays. We initially picked 30 µM and 100 µM concentrations of $FeCl_2$ and $FeCl_3$, which are considered iron-replete conditions. As a result, we observed that only 100 µM $FeCl_3$ had a very minor but positive impact on growth in the presence of ace-K. However, after further increasing the $FeCl_3$ concentration to 250 µM and 500 µM in the presence of ace-K, we could not see a dose-dependent response, suggesting the role of iron uptake in protection against ace-K is not significant (Supplementary Figure S5). This is now discussed in lines 231-246. We also acknowledge that some aspects of this section had too much description so we have now trimmed it down to describe only relevant, functionally related groups of genes.

Lines 309-311. It would be useful to give some examples of the genes that are being referred to and why this is indicative of membrane stress. How do these gene profiles fit with other membrane stressors, particularly EDTA which also causes membrane permeabilization via cation destabilisation.

We really appreciate this reviewer's suggestion here and agree it would be very useful to the reader. Therefore, we have included an additional table (Supplementary Table S3). This table highlights the high proportion of membrane protein coding genes significantly regulated in our experimental conditions, after filtering them using the annotation gene ontology obtained from FUNAGE-Pro.

We did not observe an interpretable overlap with genes differentially regulated by other membrane stressors. However, we sought to provide further insights on the impact of ace-K on the cell envelope, so we now have an entire additional section in the results that sheds more light on the impact on the membrane. Specifically, we tested the effect of ace-K on the membrane by staining it with the membrane-specific dye nonyl acridine orange (NAO) and monitoring its integrity at different time points using live cell imaging. Although cells initially presented an uneven NAO staining pattern, suggesting a dedicated membrane organisation, the NAO signal became diffuse after ace-K treatment, strongly indicating a disruption of the membrane homeostasis (Figure 6ab). Using live cell imaging, we were also able to determine that cells rapidly lose their native morphology and bulges appear in their membrane, prior to cell lysis. These findings are now discussed in lines 351-387.

The wound model data are not hugely convincing and would benefit from a comparator treatment. How does Ace-K compare to alternative wound cleaning antiseptics? Alternatively, does Ace-K potentiate antibiotics in the context of the wound model?
We have taken on board the reviewers insightful comments and have reperformed the *ex vivo* wound model assays. As a comparator, we used an industry leading wound antiseptic, Sterets Unisept whose active ingredient is chlorhexidine, as suggested by the reviewer. In parallel assays, we show that in the *ex vivo* model that ace-k is more effective in reducing the number of viable, biofilm bound cells (1.9 log reduction) than the Unisept (1.3 log reduction) (Figure 9a). This highlights the potential of ace-K which when combined with the previous biofilm disruption data (Supplementary Figure S4) suggests that a ace-K has the potential to treat chronic wounds, which are a major burden to healthcare systems globally. This is now discussed in lines 443-449.

Polymyxins have been used topically to treat wound infections in formulations such as Neosporin. Polymyxin B activity has also been shown to be potentiated in the presence of ace-K (Figure 7a). Therefore, we chose to assess the ability of ace-K to potentiate the activity of polymyxin B in our *ex vivo* model. This assay demonstrated that the use of polymyxin B in combination with ace-K lead to a greater reduction in viable bacterial numbers compared to either treatment alone (Figure 9b). This is now discussed in lines 450-459.

It appears that Ace-K is destabilising the membrane by disrupting the cation bridges between LPS molecules, most likely by chelating cations (since growth was restored by cation supplementation). How will this impact wound healing since cations are required for the integrity of cell-cell interactions and attachment to the connective tissue?
The reviewer raises an interesting point here. It has been shown that the levels of cations at a wound site are highly variable depending on the stage of wound healing, with calcium and magnesium both varying widely over a five-day period post wounding (Lansdown et al., 1999 PMID: 10580852; Coger et al., 2019, PMID: 30552609). Therefore, it is very unlikely that a single 60-minute treatment, even one applied daily, will have a significant impact on wound healing in the long term.

Minor points:

Lines 66-70 is certainly true but would benefit from supporting references.
We have modified the text accordingly.

Lines 94-97 are a little confusing. If phytochemicals are promising, why look elsewhere? Why not develop the phytochemicals?
The authors acknowledge the utility of phytochemicals with antimicrobial and anti-virulence properties. However, these have been extensively studied and well characterised. For artificial sweeteners there is a dearth of information and evidence regarding their antimicrobial activity, anti-virulence properties, and their potential as a clinical wound therapy. It is our hope that through this

study it becomes clear that, while compounds like phytochemicals and currently used drugs represent an extensive reservoir of potential new antimicrobials, due consideration should be given to other potential sources like commonly used food additives such as artificial sweeteners.

Line 103 - what is meant by 'intensive'?
We have modified the text accordingly.

Line 141: remove 'the'.
We have modified the text accordingly.

Figure 2. The text describing figure 2 falls into two sections with separate headings. Therefore, I would either merge the text or split the figure to make it easier to follow for the reader.
We have modified the text accordingly.

Lines 325-328. I don't understand why the authors find it remarkable that Ace-K inhibits the growth of multiple species of bacteria, since they have already shown an effect on two different species and effects have been reported already in other bacteria.
We appreciate the reviewers comment here and have removed the word remarkable.

Referee #3 (Remarks for Author):

This study shows that artificial sweeteners could show an inhibitory effect on the growth and virulence of infectious pathogens. This is a novel and interesting finding. Here are some concerns.

1. Most of all studies are focused on the Ace-K but the saccharin also has a strong inhibitory effect on the growth of bacteria. Does saccharin have a similar effect as Ace-K on mobility, biofilm formation, and membrane stability? Some key experiments should be done to address for making clear that these effects are specific for Ace-K or for all artificial sweeteners?
We appreciate the reviewers' comments here. We focused this study on the effect of ace-K as this artificial sweetener demonstrated the greatest inhibitory effect against both *A. baumannii* and *P. aeruginosa*. We have updated the text to offer more clarity as to why the focus was placed on ace-K, Lines 170-174. It is true that sodium saccharin showed a greater effect against *A. baumannii* than ace-K. However, we believe that due to the major structural differences between ace-K and sodium saccharin that they will have a different mechanism of action. Therefore, a full assessment of the mechanisms of action of sodium saccharin and sodium cyclamate will be the focus of future studies.

2. It is very interesting to see a positive result in the ex vivo model, however, since there is lacking a positive control (an antibiotic). So, it is too difficult to compare the results to see whether it is feasible to be applied in clinical in the future. Also, the dose of 8.85% seems to be a very high dose and was not tested in previous studies (in inhibitory effect, mobility, biofilm).
We appreciate the reviewers' clinical insight here and have sought to address this comment by performing additional experiments. We have now included a suitable positive control tested in our *ex vivo* model, the commercial wound antiseptic, Unisept, which contains chlorhexidine as an active ingredient. We show that ace-k is more effective, yielding a 1.9 log reduction in viable cells, while Unisept treatment resulted in 1.3 log reduction in viable biofilm bound cells (Figure 9a). Gauze dressings were loaded with 8.85% ace-K as this concentration was shown to be effective in dispersing biofilm (Supplementary Figure S4). This additional data is now discussed in lines 443-449. Furthermore, the wound dressings were overloaded to ensure an effective concentration of ace-k was maintained at the site of infection. We believe this additional data significantly strengthens the manuscript and we again thank the reviewer for this suggestion.

3. Why Ace-K could destabilize the membrane, what are possible explanations? Will it affect mammalian cell membrane stability?

The reviewer raises an excellent point with respect to determining how ace-K is influencing the membrane. To address this question, we performed a number of additional experiments which we believe now give considerable novel insights into how the membrane responds to ace-K exposure. We first sought to confirm a loss in membrane integrity in the presence of sub-MIC concentrations of ace-k (1.33%). Using differential fluorescent staining with both Nile red (membrane specific dye) and DAPI (chromosomal specific dye) we demonstrate that AB5075 exposed to 1.33% ace-k has an increased membrane permeability as indicated by the increase in chromosomal staining by DAPI. This high level of chromosomal staining was not apparent in control samples indicating a less permeable membrane (Supplementary Figure S8). The text has now been updated to reflect this additional data in lines 357-363.

We then sought to further address this comment by visualising these changes in the membrane in real time using time lapse microscopy. It was observed that *A. baumannii* cells stop dividing and lose structural integrity, swelling in size rapidly, upon ace-K exposure. We also observed the formation of bulges in the bacterial cell. Using the Cardiolipin (CL)-specific fluorescent dye 10-N-nonyl-acridine orange (NAO) to visualise CL distribution, we could see clear structural rearrangements in the phospholipid composition of the cell membrane and we could also confirm that the bulges were evaginating from cells (Fig. 6a, Supplementary Movie 1 & 2). We repeated our live cell imaging using the carbapenem resistant *E. coli* NCTC 1347 and observed a conserved loss of morphology, but distinct from that seen in *A. baumannii*, in that instead of cells swelling, *E. coli* cells filamented, extending to many times their original size before eventually, forming characteristic membrane bulges and ultimately lysing (Figure 6b). We performed live imaging microscopy of an *E. coli* MG1655 strain with labelled mCherry-Fis and CFP-FtsZ to understand where these bulges are emanating from and their contents. Fis is a small DNA-binding region that binds to a large number of regions of the chromosome, allowing the visualisation of the nucleosome in living cells. FtsZ is a component of the Z ring, showing future cell division sites. This time lapse experiment revealed that the membrane bulges were largely localised to either a site where a septum is formed or at a site where invagination has already taken place. (Figure 6cd, Supplementary Movie 3-5) The mCherry-Fis also confirmed that these bulges contain nuclear material. This suggests that the mechanism through which ace-k is leading to cell death is through bulge mediated cell lysis. These findings are discussed in lines 363-387. We again, thank the reviewer for this comment as we feel in addressing it, we have significantly added to our understanding of how ace-K impacts the cell. Given the extensive health and safety data available on ace-K, we do next expect that it will impact mammalian membrane stability.

4. What are IC50s for each sweetener on inhibitory effect? these should be labelled on the Figures.

We did not perform dose response experiments to determine the IC_{50} for all of the sweeteners as we chose to focus this study on the effects of ace-K, given its potency. However, we have now added the details of the IC_{50} for ace-K in lines 183-187.

5. Some functional experiments are needed for dRNA-seq validation. The authors found the pathways were responsible for the Ace-K effect. However, genetic evidence was lacking. Whether pil or com overexpression strains could resist the Ace-K effect?

The reviewer raises a valid point here and one we have addressed through additional functional experiments to validate the RNA-Seq results. Specifically, we have validated the reduced expression of *pilA,* using a transcriptional fusion reporter. *A. baumannii* AB5075 bearing the *PpilA::gfp* fusion

produced decreasing levels of fluorescence over increasing concentrations of ace-K, thus validating our dRNA-seq results (Figure 4b). Given the well-established role of the T4P in twitching motility we believe this reduction in motility is mediated through the reduced expression of the T4P associated genes when exposed to ace-K (*pilA, fimTpilVWXY, pilGHIJL, pilZ, pilTU, pilBCD and pilR* were all down regulated in the dRNA-seq data). To functionally validate this, we performed a range of twitching motility assays. In agreement with the dRNA-seq and fluorimetry results, we observed a dose-dependent decrease in the twitching motility of AB5075 over increasing concentrations of ace-K. Furthermore, this effect was not strain-specific, as other commonly used *A. baumannii* strains (AB0057 and BAA 747) also exhibited a dose-dependent decrease in twitching motility within the same range of ace-K concentrations (Supplementary Figure S7ab). We also reperformed the transformation assays at a range of concentrations with a clear decrease in transformation frequency with increasing ace-k concentrations (Figure 4d, Supplementary Figure 7c). This data is discussed in lines 285-300.

We next assessed the *csu* pili, which have been shown to influence biofilm formation (Moon *et al.,* 2017, PMID: 28674047, Luo *et al.,* 2015, PMID: 25888221, Pakharukova *et al.,* 2018, PMID: 29735695). We confirmed the link between the *csu* pili and biofilm formation under our conditions by confirming that transposon mutants in *csuA/B, csuC* and *csuE* (all down regulated significantly in our RNA-Seq data set) all have diminished biofilm formation (Supplementary Figure S6) supporting the hypothesis that ace-K is disrupting biofilm through the downregulation of the *csu* pili. This data is discussed in lines 248-263.

Finally, as outlined in the previous point, given the large number of membrane related coding genes dysregulated (Supplementary Table S3), we have confirmed that indeed the membrane is severely impacted by ace-K exposure. We did not attempt to overexpress *com* or *pil* genes and measure ace-K sensitivity, as the additional experiments performed to address the previous comments suggest that the cell lysis effects of ace-K are independent of pili.

6. Whether it is possible for testing the Ace-K role of sensitizing MRS to antibiotics in an animal model?

We agree with the reviewer that showing the resensitizing effect of ace-k in an *in vivo* model would be of significant interest however live animal experiments would be beyond the remit of this current manuscript but will be the focus of future work. However, based on this suggestion and the suggestion of the other reviewer we did perform additional *ex vivo* assays to determine if ace-k could influence antibiotic potency in this model. Polymyxin B activity has also been shown to be potentiated in the presence of ace-K (Figure 7a). Therefore, we chose to assess the ability of ace-K to potentiate the activity of polymyxin B in our *ex vivo* model. This assay demonstrated that the use of polymyxin B in combination with ace-K lead to a greater reduction in viable bacterial numbers compared to either treatment alone (Figure 9b). This is now discussed in lines 450-459.

27th Oct 2022

Dear Dr. McCarthy,

Thank you for the submission of your revised manuscript to EMBO Molecular Medicine. I am pleased to inform you that we will be able to accept your manuscript pending the following final amendments:

1) In the main manuscript file, please do the following:
- There is still a callout for supplementary Table S5. Please correct.
- In M&M, add statistical paragraph that should reflect all information that you have filled in the Authors Checklist, especially regarding randomization, blinding, replication.
- Data availability: In addition to the accession number please provide URL for deposited datasets. Please be aware that all datasets should be made freely available upon acceptance, without restriction. Use the following format to report the accession number of your data:

The datasets produced in this study are available in the following databases:
[data type]: [full name of the resource] [accession number/identifier] ([doi or URL or identifiers.org/DATABASE:ACCESSION])

Please check "Author Guidelines" for more information.
https://www.embopress.org/page/journal/17574684/authorguide#availabilityofpublishedmaterial
2) For more information: This space should be used to list relevant web links for further consultation by our readers. Could you identify some relevant ones and provide such information as well? Some examples are patient associations, relevant databases, OMIM/proteins/genes links, author's websites, etc...
3) As part of the EMBO Publications transparent editorial process initiative (see our Editorial at http://embomolmed.embopress.org/content/2/9/329), EMBO Molecular Medicine will publish online a Review Process File (RPF) to accompany accepted manuscripts. This file will be published in conjunction with your paper and will include the anonymous referee reports, your point-by-point response and all pertinent correspondence relating to the manuscript. Let us know whether you agree with the publication of the RPF and as here, if you want to remove or not any figures from it prior to publication. Please note that the Authors checklist will be published at the end of the RPF.
4) Please provide a point-by-point letter INCLUDING my comments as well as the reviewer's reports and your detailed responses (as Word file).

I look forward to reading a new revised version of your manuscript as soon as possible.

Yours sincerely,

Zeljko Durdevic

***** Reviewer's comments *****

Referee #1 (Comments on Novelty/Model System for Author):

This is a high-quality pre-clinical study.

Referee #1 (Remarks for Author):

The authors have undertaken significant additional experimental work, as well as extensive edits of the text that fully address my concerns.

No further points to raise.

Referee #2 (Remarks for Author):

Is suitable for publication

The authors addressed the minor editorial issues.

We are pleased to inform you that your manuscript is accepted for publication and is now being sent to our publisher to be included in the next available issue of EMBO Molecular Medicine.

EMBO Press Author Checklist

Corresponding Author Name: Ronan McCarthy	
Journal Submitted to: EMBO Molecular Medicine	
Manuscript Number: EMM-2022-16397-T	

USEFUL LINKS FOR COMPLETING THIS FORM
The EMBO Journal - Author Guidelines
EMBO Reports - Author Guidelines
Molecular Systems Biology - Author Guidelines
EMBO Molecular Medicine - Author Guidelines

Reporting Checklist for Life Science Articles (updated January 2022)
This checklist is adapted from Materials Design Analysis Reporting (MDAR) Checklist for Authors. MDAR establishes a minimum set of requirements in transparent reporting in the life sciences (see Statement of Task: 10.31222/osf.io/9sm4x). Please follow the journal's guidelines in preparing your manuscript.
Please note that a copy of this checklist will be published alongside your article.

Abridged guidelines for figures
1. Data
The data shown in figures should satisfy the following conditions:
- the data were obtained and processed according to the field's best practice and are presented to reflect the results of the experiments in an accurate and unbiased manner.
- ideally, figure panels should include only measurements that are directly comparable to each other and obtained with the same assay.
- plots include clearly labeled error bars for independent experiments and sample sizes. Unless justified, error bars should not be shown for technical replicates.
- if n<5, the individual data points from each experiment should be plotted. Any statistical test employed should be justified.
- Source Data should be included to report the data underlying figures according to the guidelines set out in the authorship guidelines on Data Presentation.

2. Captions
Each figure caption should contain the following information, for each panel where they are relevant:
- a specification of the experimental system investigated (eg cell line, species name).
- the assay(s) and method(s) used to carry out the reported observations and measurements.
- an explicit mention of the biological and chemical entity(ies) that are being measured.
- an explicit mention of the biological and chemical entity(ies) that are altered/varied/perturbed in a controlled manner.
- the exact sample size (n) for each experimental group/condition, given as a number, not a range;
- a description of the sample collection allowing the reader to understand whether the samples represent technical or biological replicates (including how many animals, litters, cultures, etc.).
- a statement of how many times the experiment shown was independently replicated in the laboratory.
- definitions of statistical methods and measures:
  - common tests, such as t-test (please specify whether paired vs. unpaired), simple χ2 tests, Wilcoxon and Mann-Whitney tests, can be unambiguously identified by name only, but more complex techniques should be described in the methods section;
  - are tests one-sided or two-sided?
  - are there adjustments for multiple comparisons?
  - exact statistical test results, e.g., P values = x but not P values < x;
  - definition of 'center values' as median or average;
  - definition of error bars as s.d. or s.e.m.

Please complete ALL of the questions below.
Select "Not Applicable" only when the requested information is not relevant for your study.

Materials

Newly Created Materials	Information included in the manuscript?	In which section is the information available? (Reagents and Tools Table, Materials and Methods, Figures, Data Availability Section)
New materials and reagents need to be available; do any restrictions apply?	Yes	Reporter strains available upo request.

Antibodies	Information included in the manuscript?	In which section is the information available? (Reagents and Tools Table, Materials and Methods, Figures, Data Availability Section)
For **antibodies** provide the following information:<br>- Commercial antibodies: RRID (if possible) or supplier name, catalogue number and or/clone number<br>- Non-commercial: RRID or citation	Not Applicable	

DNA and RNA sequences	Information included in the manuscript?	In which section is the information available? (Reagents and Tools Table, Materials and Methods, Figures, Data Availability Section)
**Short novel DNA or RNA including primers, probes**: provide the sequences.	Yes	Supplementary Table 5

Cell materials	Information included in the manuscript?	In which section is the information available? (Reagents and Tools Table, Materials and Methods, Figures, Data Availability Section)
**Cell lines:** Provide species information, strain. Provide accession number in repository **OR** supplier name, catalog number, clone number, and/**OR** RRID.	Not Applicable	
**Primary cultures:** Provide species, strain, sex of origin, genetic modification status.	Not Applicable	
Report if the cell lines were recently **authenticated** (e.g., by STR profiling) and tested for mycoplasma contamination.	Not Applicable	

Experimental animals	Information included in the manuscript?	In which section is the information available? (Reagents and Tools Table, Materials and Methods, Figures, Data Availability Section)
**Laboratory animals or Model organisms:** Provide species, strain, sex, age, genetic modification status. Provide accession number in repository **OR** supplier name, catalog number, clone number, **OR** RRID.	Not Applicable	
**Animal observed in or captured from the field:** Provide species, sex, and age where possible.	Not Applicable	
Please detail **housing and husbandry conditions**.	Not Applicable	

Plants and microbes	Information included in the manuscript?	In which section is the information available? (Reagents and Tools Table, Materials and Methods, Figures, Data Availability Section)
**Plants:** provide species and strain, ecotype and cultivar where relevant, unique accession number if available, and source (including location for collected wild specimens).	Not Applicable	
**Microbes:** provide species and strain, unique accession number if available, and source.	Yes	All strain details including source is available in supplementary table 5.

Human research participants	Information included in the manuscript?	In which section is the information available? (Reagents and Tools Table, Materials and Methods, Figures, Data Availability Section)
If collected and within the bounds of privacy constraints report on age, sex and gender or ethnicity for all study participants.	Not Applicable	

Core facilities	Information included in the manuscript?	In which section is the information available? (Reagents and Tools Table, Materials and Methods, Figures, Data Availability Section)
If your work benefited from core facilities, was their service mentioned in the acknowledgments section?	Not Applicable	

Design

Study protocol	Information included in the manuscript?	In which section is the information available? (Reagents and Tools Table, Materials and Methods, Figures, Data Availability Section)
If study protocol has been **pre-registered, provide DOI in the manuscript**. For clinical trials, provide the trial registration number **OR** cite DOI.	Not Applicable	
Report the **clinical trial registration number** (at ClinicalTrials.gov or equivalent), where applicable.	Not Applicable	

Laboratory protocol	Information included in the manuscript?	In which section is the information available? (Reagents and Tools Table, Materials and Methods, Figures, Data Availability Section)
Provide DOI OR other citation details if **external detailed step-by-step protocols** are available.	Yes	All protocols are cited in the methods section.

Experimental study design and statistics	Information included in the manuscript?	In which section is the information available? (Reagents and Tools Table, Materials and Methods, Figures, Data Availability Section)
Include a statement about **sample size** estimate even if no statistical methods were used.	Yes	
Were any steps taken to minimize the effects of subjective bias when allocating animals/samples to treatment (e.g. **randomization procedure**)? If yes, have they been described?	Not Applicable	
Include a statement about **blinding** even if no blinding was done.	Not Applicable	
Describe **inclusion/exclusion criteria** if samples or animals were excluded from the analysis. Were the criteria pre-established?<br><br>If sample or data points were omitted from analysis, report if this was due to attrition or intentional exclusion and provide justification.	Not Applicable	
For every figure, are **statistical tests** justified as appropriate? Do the data meet the assumptions of the tests (e.g., normal distribution)? Describe any methods used to assess it. Is there an estimate of variation within each group of data? Is the variance similar between the groups that are being statistically compared?	Yes	Details on statistics included in each figure legends and in the methods section.

Sample definition and in-laboratory replication	Information included in the manuscript?	In which section is the information available? (Reagents and Tools Table, Materials and Methods, Figures, Data Availability Section)
In the figure legends: state number of times the experiment was **replicated** in laboratory.	Yes	
In the figure legends: define whether data describe **technical or biological replicates**.	Yes	

Ethics

Ethics	Information included in the manuscript?	In which section is the information available? (Reagents and Tools Table, Materials and Methods, Figures, Data Availability Section)
Studies involving **human participants**: State details of **authority granting ethics approval** (IRB or equivalent committee(s), provide reference number for approval.	Not Applicable	
Studies involving **human participants**: Include a statement confirming that **informed consent** was obtained from all subjects and that the experiments conformed to the principles set out in the WMA Declaration of Helsinki and the Department of Health and Human Services Belmont Report.	Not Applicable	
Studies involving **human participants:** For publication of **patient photos**, include a statement confirming that consent to publish was obtained.	Not Applicable	
Studies involving experimental **animals**: State details of **authority granting ethics approva**l (IRB or equivalent committee(s), provide reference number for approval. Include a statement of compliance with ethical regulations.	Not Applicable	
Studies involving **specimen and field samples:** State if relevant **permits** obtained, provide details of authority approving study; if none were required, explain why.	Not Applicable	

Dual Use Research of Concern (DURC)	Information included in the manuscript?	In which section is the information available? (Reagents and Tools Table, Materials and Methods, Figures, Data Availability Section)
Could your study fall under dual use research restrictions? Please check biosecurity documents and list of **select agents and toxins** (CDC): https://www.selectagents.gov/sat/list.htm	Not Applicable	
If you used a select agent, is the security level of the lab appropriate and reported in the manuscript?	Not Applicable	
If a study is subject to dual use research of concern regulations, is the name of the **authority granting approval and reference number** for the regulatory approval provided in the manuscript?	Not Applicable	

Reporting

The MDAR framework recommends adoption of discipline-specific guidelines, established and endorsed through community initiatives. Journals have their own policy about requiring specific guidelines and recommendations to complement MDAR.

Adherence to community standards	Information included in the manuscript?	In which section is the information available? (Reagents and Tools Table, Materials and Methods, Figures, Data Availability Section)
State if relevant guidelines or checklists (e.g., **ICMJE, MIBBI, ARRIVE, PRISMA**) have been followed or provided.	Not Applicable	
For **tumor marker prognostic studies,** we recommend that you follow the **REMARK** reporting guidelines (see link list at top right). See author guidelines, under 'Reporting Guidelines'. Please confirm you have followed these guidelines.	Not Applicable	
For **phase II and III randomized controlled trials**, please refer to the **CONSORT** flow diagram (see link list at top right) and submit the CONSORT checklist (see link list at top right) with your submission. See author guidelines, under 'Reporting Guidelines'. Please confirm you have submitted this list.	Not Applicable	

Data Availability

Data availability	Information included in the manuscript?	In which section is the information available? (Reagents and Tools Table, Materials and Methods, Figures, Data Availability Section)
Have **primary datasets** been deposited according to the journal's guidelines (see 'Data Deposition' section) and the respective accession numbers provided in the Data Availability Section?	Yes	Data Availability Section
Were **human clinical and genomic datasets** deposited in a public access-controlled repository in accordance to ethical obligations to the patients and to the applicable consent agreement?	Not Applicable	
Are **computational models** that are central and integral to a study available without restrictions in a machine-readable form? Were the relevant accession numbers or links provided?	Not Applicable	
If publicly available data were reused, provide the respective **data citations in the reference list**.	Not Applicable	
